# Author Correction: Flexibility of intrinsically disordered degrons in AUX/IAA proteins reinforces auxin co-receptor assemblies

**DOI:** 10.1038/s41467-021-22151-x

**Published:** 2021-03-15

**Authors:** Michael Niemeyer, Elena Moreno Castillo, Christian H. Ihling, Claudio Iacobucci, Verona Wilde, Antje Hellmuth, Wolfgang Hoehenwarter, Sophia L. Samodelov, Matias D. Zurbriggen, Panagiotis L. Kastritis, Andrea Sinz, Luz Irina A. Calderón Villalobos

**Affiliations:** 1 Molecular Signal Processing Department, Leibniz Institute of Plant Biochemistry (IPB), Weinberg 3, 06120 Halle (Saale), Germany; 2 Department of Pharmaceutical Chemistry & Bioanalytics, Institute of Pharmacy, Martin Luther University Halle-Wittenberg, Charles Tanford Protein Center, Kurt-Mothes-Straße 3a, 06120 Halle (Saale), Germany; 3 Proteome Analytics, Leibniz Institute of Plant Biochemistry (IPB), Weinberg 3, 06120 Halle (Saale), Germany; 4 Institute of Synthetic Biology & Cluster of Excellence on Plant Science (CEPLAS), Heinrich-Heine University of Düsseldorf, Universitätsstrasse 1, 40225 Düsseldorf, Germany; 5 ZIK HALOMEM & Institute of Biochemistry and Biotechnology, Martin Luther University Halle-Wittenberg, Biozentrum, Weinbergweg 22, 06120 Halle (Saale), Germany

**Keywords:** Biochemistry, Nuclear receptors, Biophysics, Plant sciences

Correction to: *Nature Communications* 10.1038/s41467-020-16147-2, published online 8 May 2020.

The original version of this Article incorrectly reported *K*_d_ values in the fourth and fifth sentences of the second paragraph of the ‘Intrinsic disorder impacts auxin-driven receptor association’ section of the Results, which incorrectly read ‘We observed the general trend of reduced auxin binding affinities when altering IAA7 in TIR1·IAA7BM3 (*K*_d_ = ~53 ± 2 nM), TIR1·IAA (7-7-7-12-7) (*K*_d_ = 93  nM), and TIR1·IAA (7-7-7-7-12) (*K*_d_ = 86  ± 25 nM) complexes in comparison to TIR1·IAA(7-7-7-7-7) (*K*_d_ = ~20 ± 6 nM) (*n* = 2–5; ± indicates SEM). We, however, measured relatively similar auxin affinities of TIR1·IAA12BM3 (*K*_d_ = ~143 ± 3 nM), TIR1·IAA (12-12-12-7-12) (*K*_d_ = ~172 ±  76 nM), TIR1·IAA (12-12-12-12-7) (*K*_d_ = ~152 ± 63 nM), and TIR1·IAA12 (*K*_d_ =  ~224 ± 66 nM); (*n* = 2–5; ± indicates SEM) (Fig. 2b, c and Supplementary Fig. 6.)’

The correct version states ‘TIR1·IAA (7-7-7-12-7) (*K*_d_ = ~57 ± 2 nM)’ in place of ‘TIR1·IAA (7-7-7-12-7) (*K*_d_ = 93 nM)’, ‘TIR1·IAA (7-7-7-7-12) (*K*_d_ = ~43 ±  12 nM)’ in place of ‘TIR1·IAA (7-7-7-7-12) (*K*_d_ = 86 ± 25 nM)’, ‘TIR1·IAA(7-7-7-7-7) (*K*_d_ = ~20 ± 3nM)’ in place of ‘TIR1·IAA(7-7-7-7-7) (*K*_d_ = ~20 ± 6 nM)’, ‘TIR1·IAA (12-12-12-7-12) (*K*_d_ = ~79 ± 22 nM)’ in place of ‘TIR1·IAA (12-12-12-7-12) (*K*_d_ = ~172 ± 76 nM)’, ‘TIR1·IAA (12-12-12-12-7) (*K*_d_ = ~133 ± 37 nM)’ in place of ‘TIR1·IAA (12-12-12-12-7) (*K*_d_ = ~152 ± 63 nM)’, and ‘TIR1·IAA12 (*K*_d_ = ~226 ± 34 nM)’ in place of ‘TIR1·IAA12 (*K*_d_ = ~224 ± 66  nM)’.

The values reported in Fig. 2 and Supplementary Fig. 6 were correct in the original version.

This has been corrected in both the PDF and HTML versions of the Article.

