# Supplementary material for: Flexibility of intrinsically disordered degrons in AUX/IAA proteins reinforces auxin co-receptor assemblies
Source: Nat Commun. 2020 May 8;11:2277. doi: 10.1038/s41467-020-16147-2 (PMC7210949; doi:10.1038/s41467-020-16147-2)
Supplement: Supplementary file 1 — Supplementary Information [file 41467_2020_16147_MOESM1_ESM.pdf]

Supplementary Information

Flexibility of intrinsically disordered degrons in AUX/IAA proteins reinforces auxin co-receptor assemblies

Niemeyer *et al.*

Supplementary Figures 1-15

Supplementary Tables 1-3

Supplementary Methods

Supp. Fig. 1

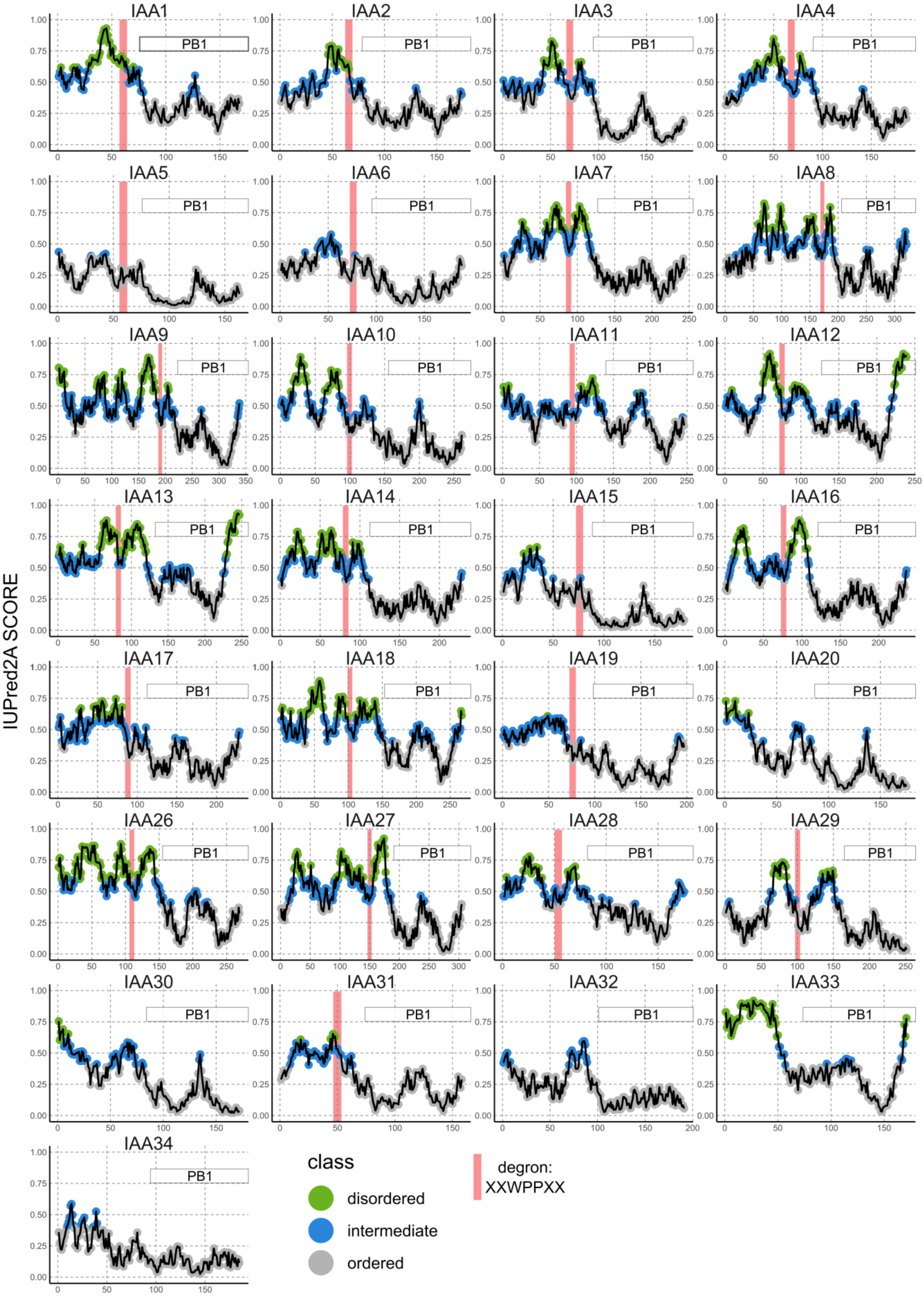

Supplementary Figure 1| IUPred2A disorder prediction along the sequence of *Arabidopsis thaliana* AUX/IAA proteins. The x-axis corresponds to the full length of each AUX/IAA protein sequence, and the y-axis shows the IUPred2A score for each amino acid (probability between 0-1). Amino acid residues are colored according to their disorder probability (disordered: ≥ 0.6, green; intermediate: 0.4-0.6, blue and ordered: ≤0.4, gray). The resolved, ordered PB1 domain is located along the sequence, as indicated, starting with the conserved VKV motif.

Supp. Fig. 2

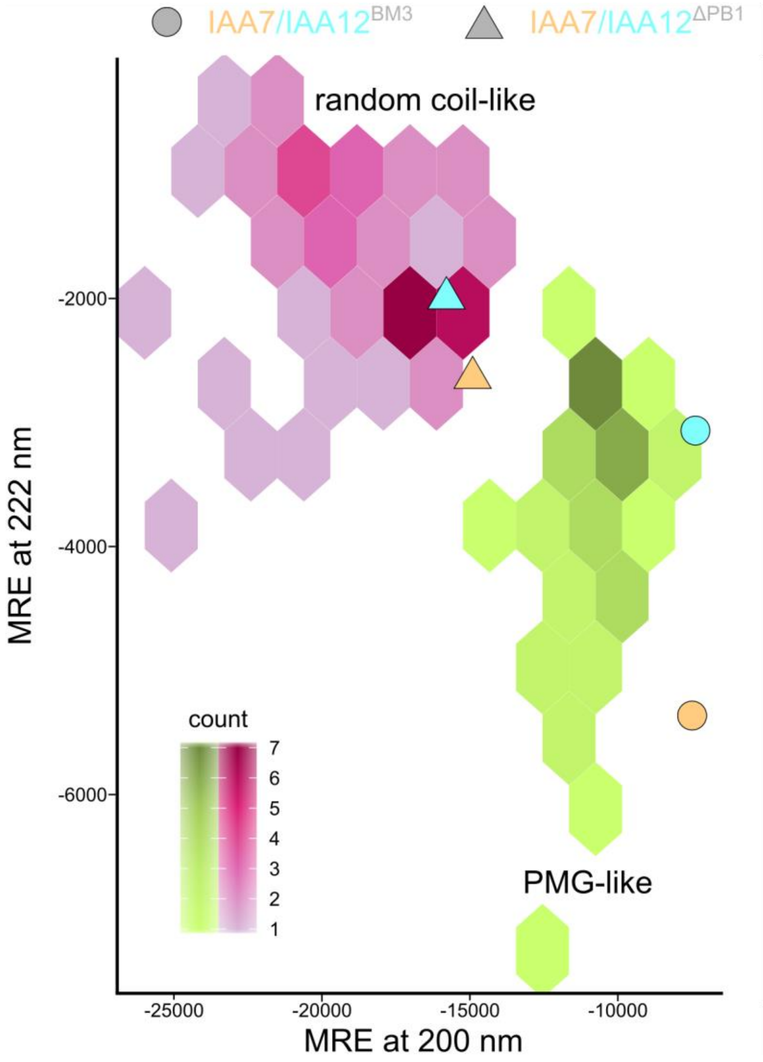

Supplementary Figure 2| Classification of IAA7 and IAA12 variants according to their CD spectra. CD spectral data classifies IAA7 (light orange) and IAA12 (aquamarine) as PMG-like proteins with random coil elements in their N-terminal half[1-3]. Molar residual ellipticity (MRE) at 200 nm and 222 nm is shown for the specified AUX/IAA protein variants on top of hexagonal binned reference proteins, with either unfolded, random coil-like proteins (purple) or premolten globule-like (PMG-like; green) proteins. Truncated versions (triangles, ΔPB1) lack the conserved folded PB1 domain. IAA7^{BM3} and IAA12^{BM3} variants (circles) carry 3 amino acid exchanges in their PB1 domain to render them oligomerization deficient.

Supp. Fig. 3

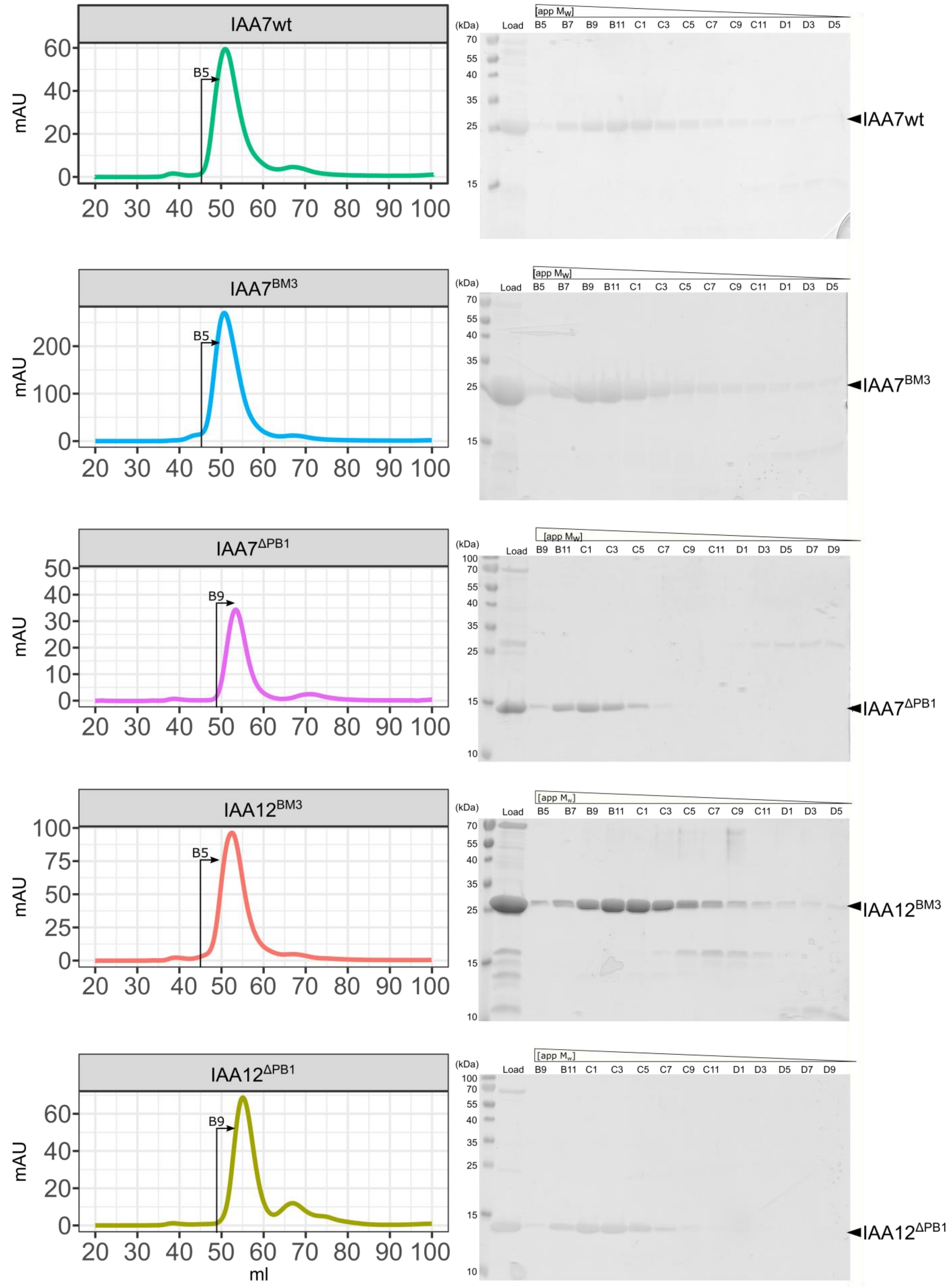

Supplementary Figure 3| Representative size exclusion chromatography runs for the untagged AUX/IAA protein variants. Elution profiles were obtained from semi-preparative size exclusion chromatography runs on a calibrated HiPrep 16/60 Sephacryl S100 High Resolution column (left panels). Indicated is the first fraction analyzed by SDS-PAGE shown in the right panels. Impurities could be separated from the protein of interest (indicated).

Supp. Fig. 4

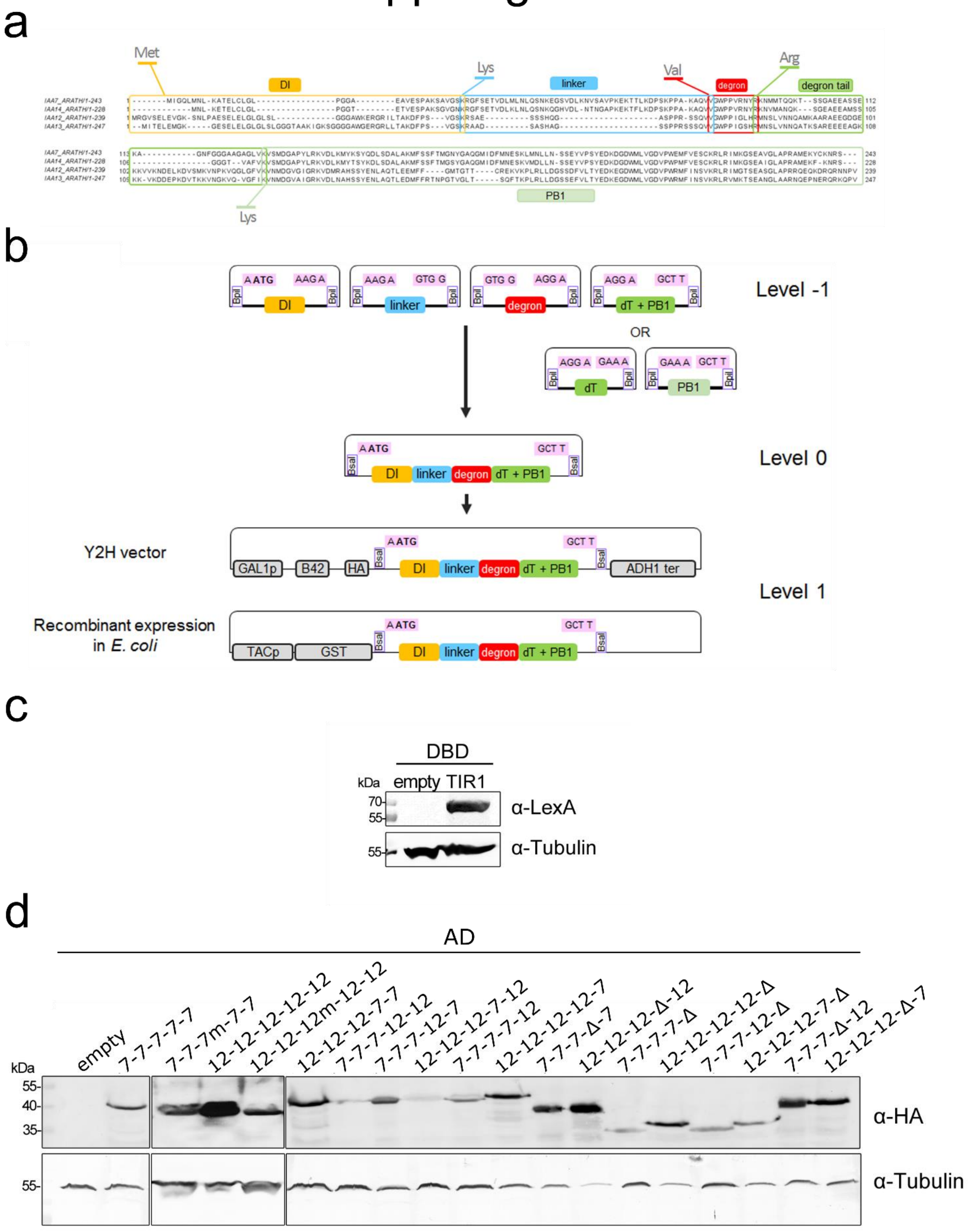

Supplementary Figure 4| Design principle for 4- and 5-module AUX/IAA chimeras. (**a**) Sequence alignment of IAA7, IAA14, IAA12, IAA13 from *Arabidopsis thaliana* showing conserved amino acid residues selected as start and end of each module: DI (orange), linker (blue), core degron (red), degron tail (dark green) and the PB1 domain (light green). Conserved amino acids used as Golden Gate assembly sites are highlighted. (**b**) Golden Gate cloning strategy to assemble level -1, 0, and 1 constructs for either yeast-two hybrid assays or recombinant *E.coli* expression as GST-fusion proteins using *Bpi*I and *Bsa*I restriction enzymes. (**c-d**) Immunoblots for LexA-DBD-tagged TIR1 (**c**) and HA-tagged AUX/IAA chimeras (**d**) from haploid yeast cells grown in Gal/Raff –Trp or Gal/Raff –Ura –His medium, respectively. 7m and 12m correspond to the gain-of-function mutations *axr2-1* and *bdl*, respectively. Detection was carried out using anti-LexA, anti-HA (F7), and anti-tubulin (loading control) antibodies.

Supp. Fig. 5

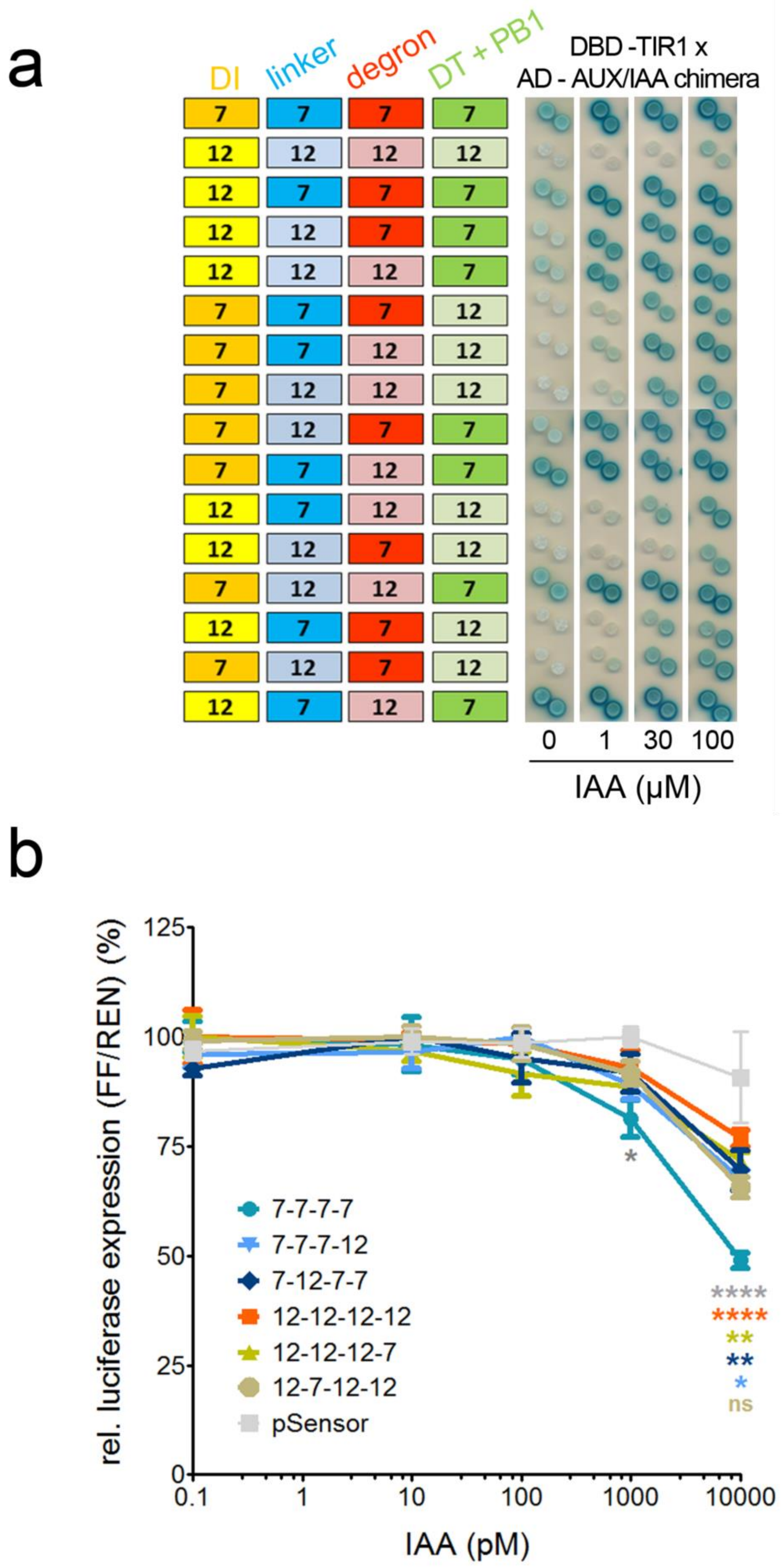

Supplementary Figure 5| Design of 4-module chimeras, where module 4 consists of the degron tail and the PB1 domain of IAA7 or IAA12 combined. (**a**) Yeast two hybrid assay shows auxin-dependent interaction of TIR1 and chimeric AUX/IAAs is strongly driven by the presence of the IAA7 degron tail, and the PB1 domain -containing module. (**b**) Ratiometric luminescent biosensor[4] to track degradation of 4-module AUX/IAA chimeric proteins in *Arabidopsis* protoplasts.

Supp. Fig. 6

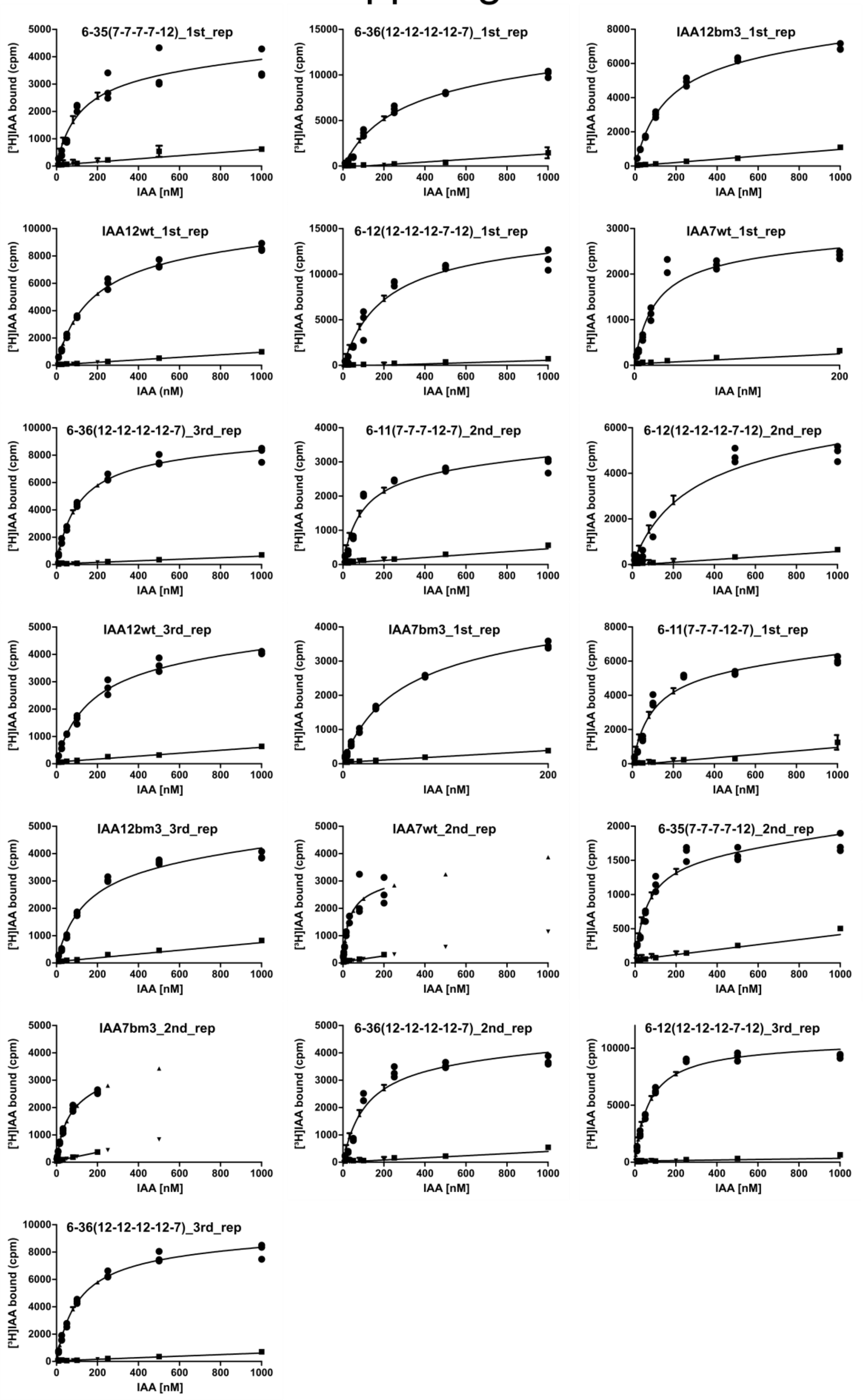

Supplementary Figure 6| Single non-normalized [^{3}H]IAA radioligand binding curves. Single binding curves for each AUX/IAA variant and chimeric construct. Datapoints of each [^{3}H]IAA concentration are shown as individual points for each technical replica (circles) together with non-specific binding in the presence of 2 mM cold IAA (squares).

Supp. Fig. 7

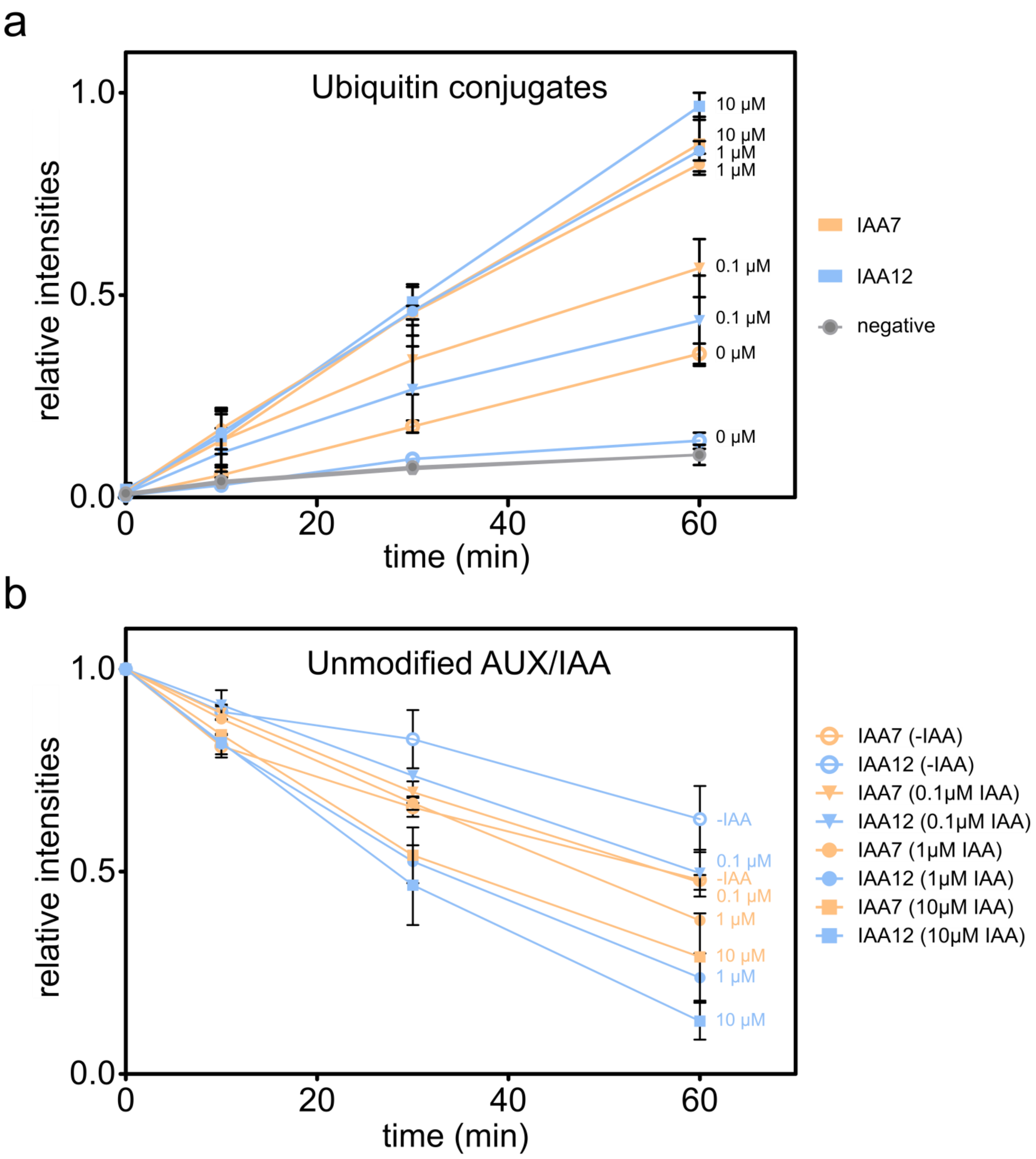

Supplementary Figure 7| Quantification of auxin- and time-dependent ubiquitylation of IAA7 and IAA12. (**a**) Increase in ubiquitin conjugates over time measured as the in-gel ubiquitin-fluorescein signal intensity above the ubiquitin-modified Cullin1 (asterisk, **Figure 3a).** Signal was normalized by the strongest signal (IAA12, 10 μM IAA). (**b**) Decrease of unmodified GST-AUX/IAA protein signal after immunoblotting detected by an Alexa Fluor Plus 647-coupled secondary antibody. Signals were normalized to the intensities at time point “0“. Depicted are mean values from three independent experiments with standard deviation as error bars. Results for GST-IAA7 and GST-IAA12 are depicted in light orange and light blue, respectively.

Supp. Fig. 8

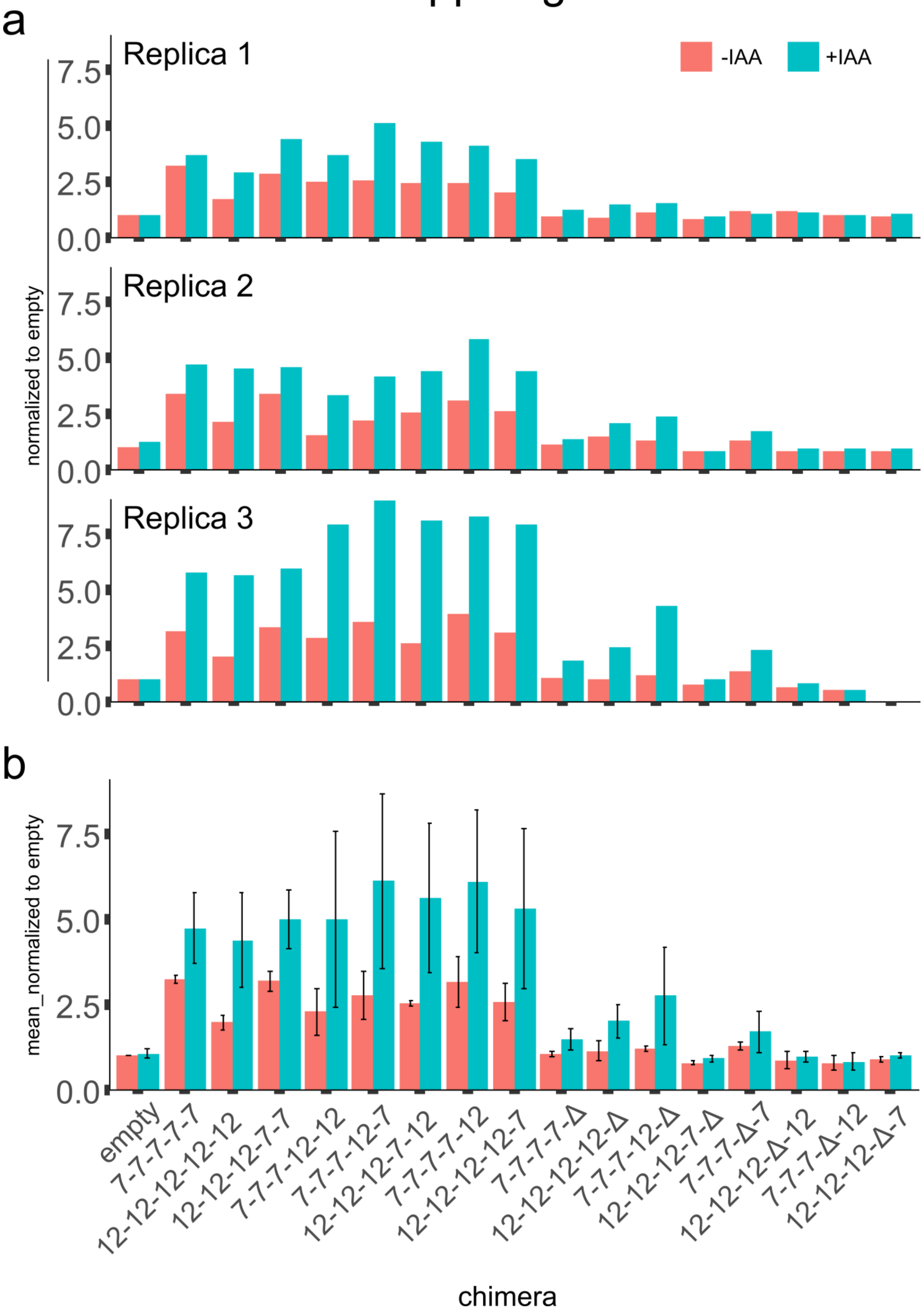

Supplementary Figure 8| Quantification of auxin-triggered chimera ubiquitylation. As in Supplementary Figure 7 ubiquitin conjugates on chimeric AUX/IAAs were measured via fluorescein signal intensities in the presence (teal) or absence (salmon) of auxin (IAA) after 1 h reaction time. (**a**) Raw signal intensities for each individual replica. (**b**) Auxin-triggered fold induction of chimera ubiquitylation as mean values with standard deviation using data from **a**. Chimeras consisting mainly of IAA7 or IAA12 modules are displayed.

Supp. Fig. 9

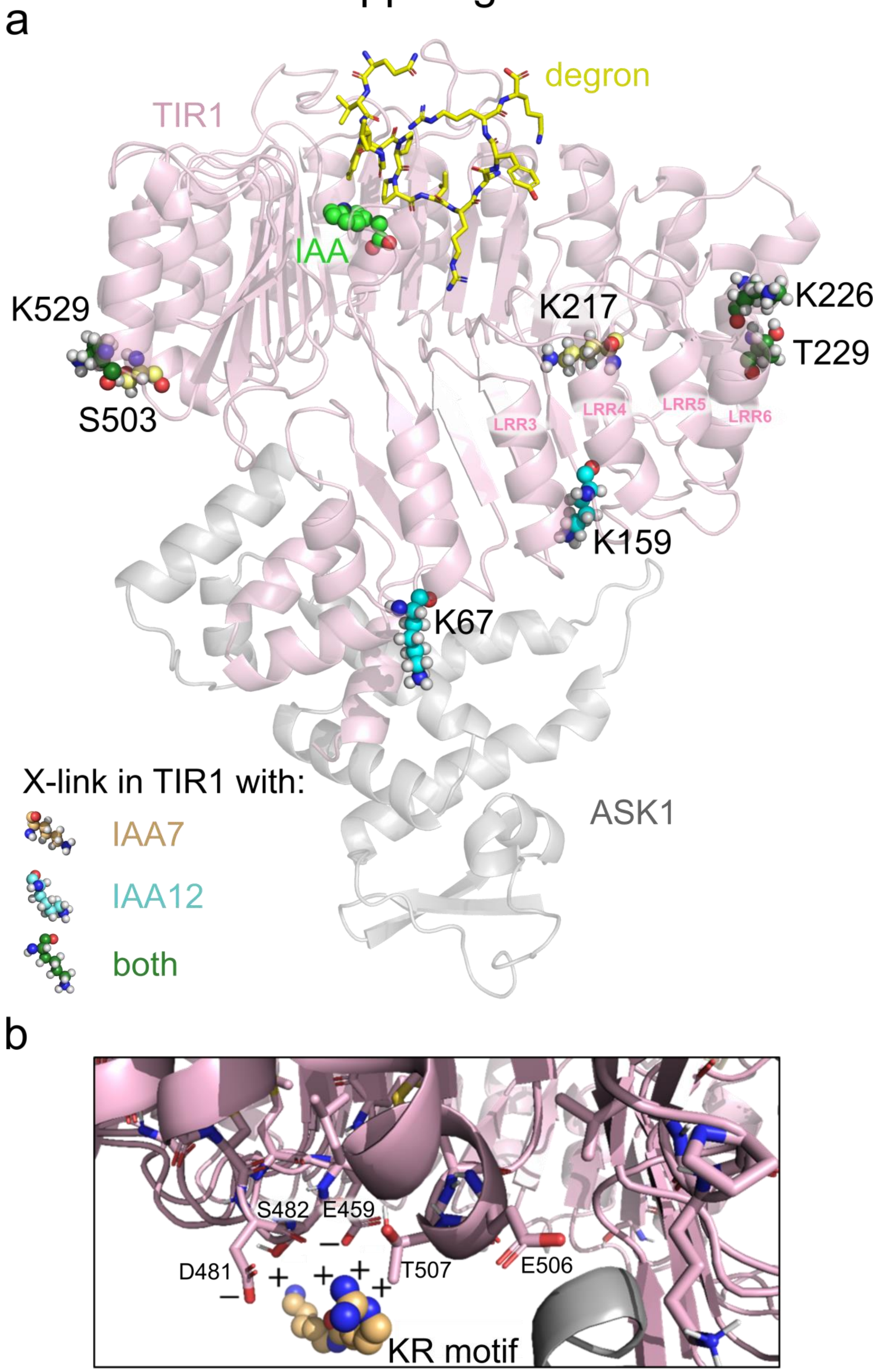

Supplementary Figure 9| Crosslinked residues in TIR1 either with IAA7, IAA12 or both. (**a**) Depicted is the crystal structure of ASK1·TIR1·auxin·IAA7 degron (2P1Q, gray, light pink) with highlighted residues found to be crosslinked with either IAA7 (light orange), IAA12 (aquamarine) or both (green) shown as spheres. Leucine-rich repeats carrying PB1 domain-interacting are labeled. (**b**) Patch enriched with negative charge potential, close to KR motif-cross-linked residues, acting as a plausible interaction site.

Supp. Fig. 10

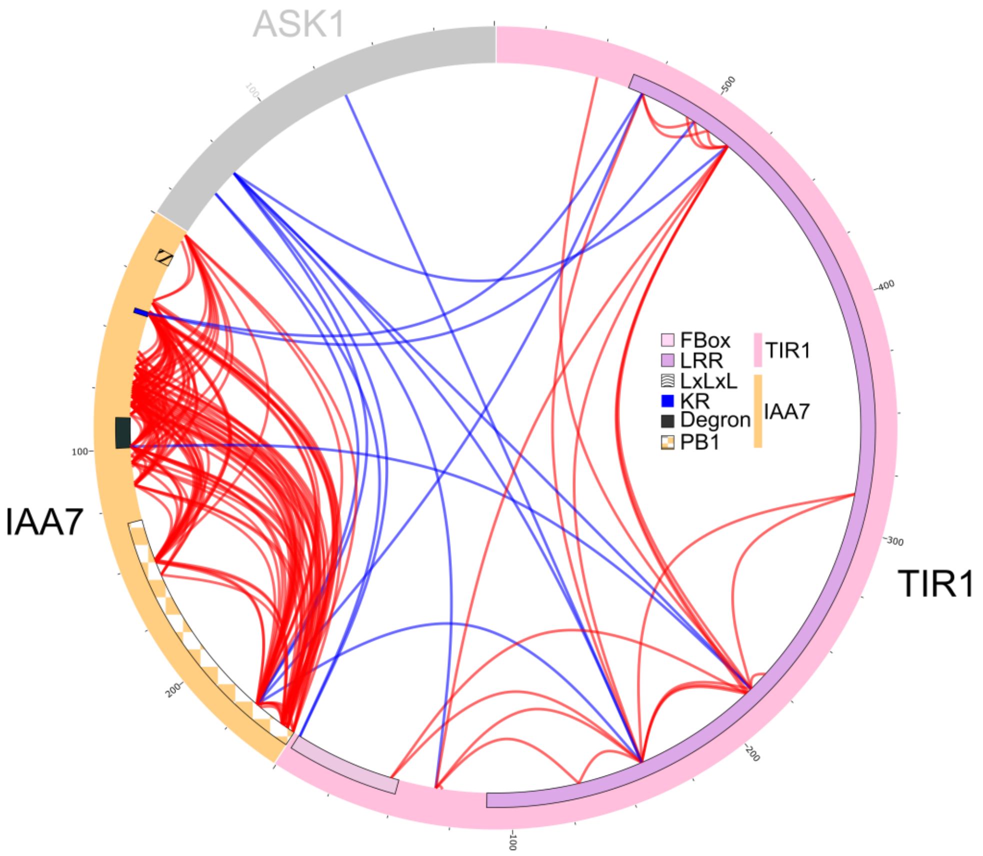

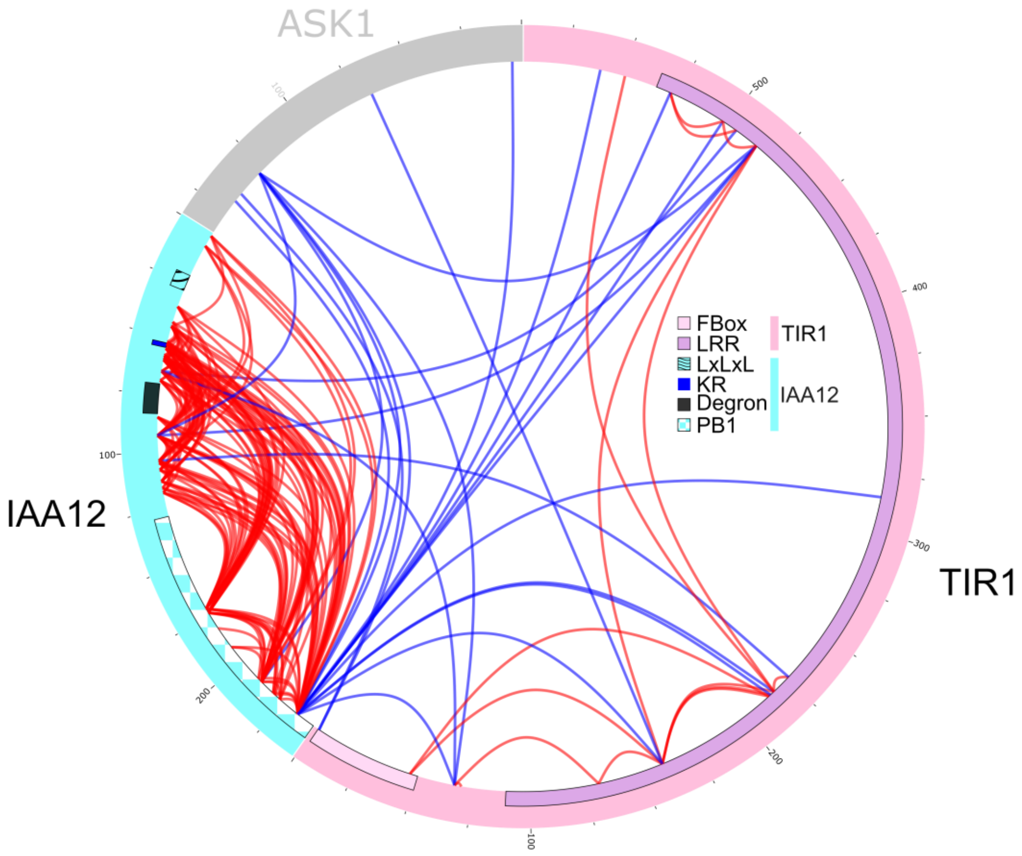

Supplementary Figure 10| Crosslinks identified in ASK1·TIR1 and AUX/IAAs in the absence of auxin. Displayed are all crosslinks within (intra-protein, red) or in between (inter-protein, blue) ASK1 (gray), TIR1 (light pink) and IAA7 (light orange) or IAA12 (aquamarine) as connecting lines along the circular depicted amino acid sequence. Lines correspond to all crosslinked peptides collected from multiple replica.

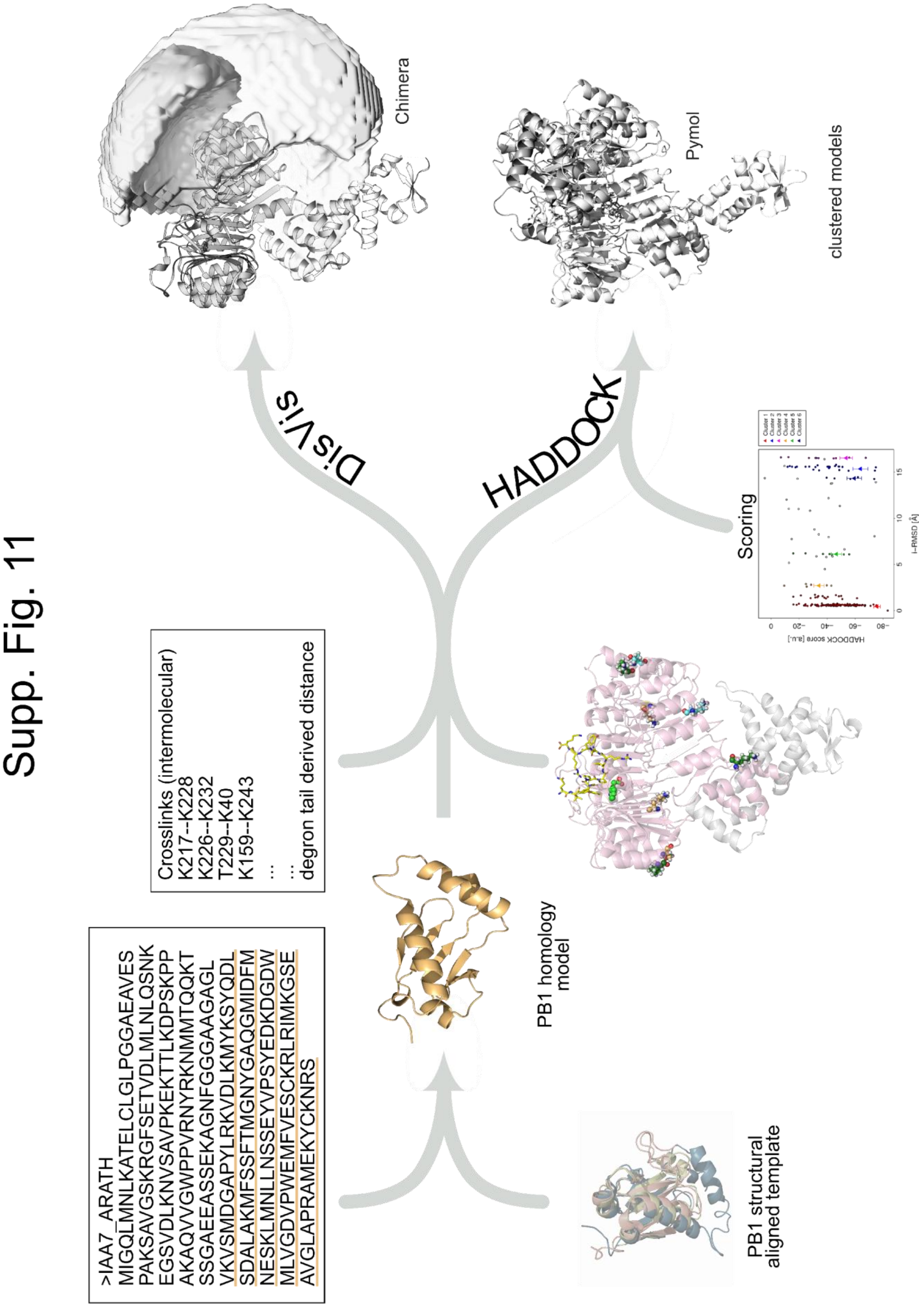

Supplementary Figure 11| Workflow for cross-linking-based docking using HADDOCK. Homology models from *At*IAA7[PB1] and *At*IAA12[PB1] domains were created using multi-template-based comparative modelling with MODELLER. Docking models using HADDOCK were generated by docking the PB1 homology models on the modified ASK1·TIR1·auxin·degron crystal structure (2P1Q) using as distant restraints the cross-linking information and the degron tail length. Potential conformational space was visualized via DisVis.

Supp. Fig. 12

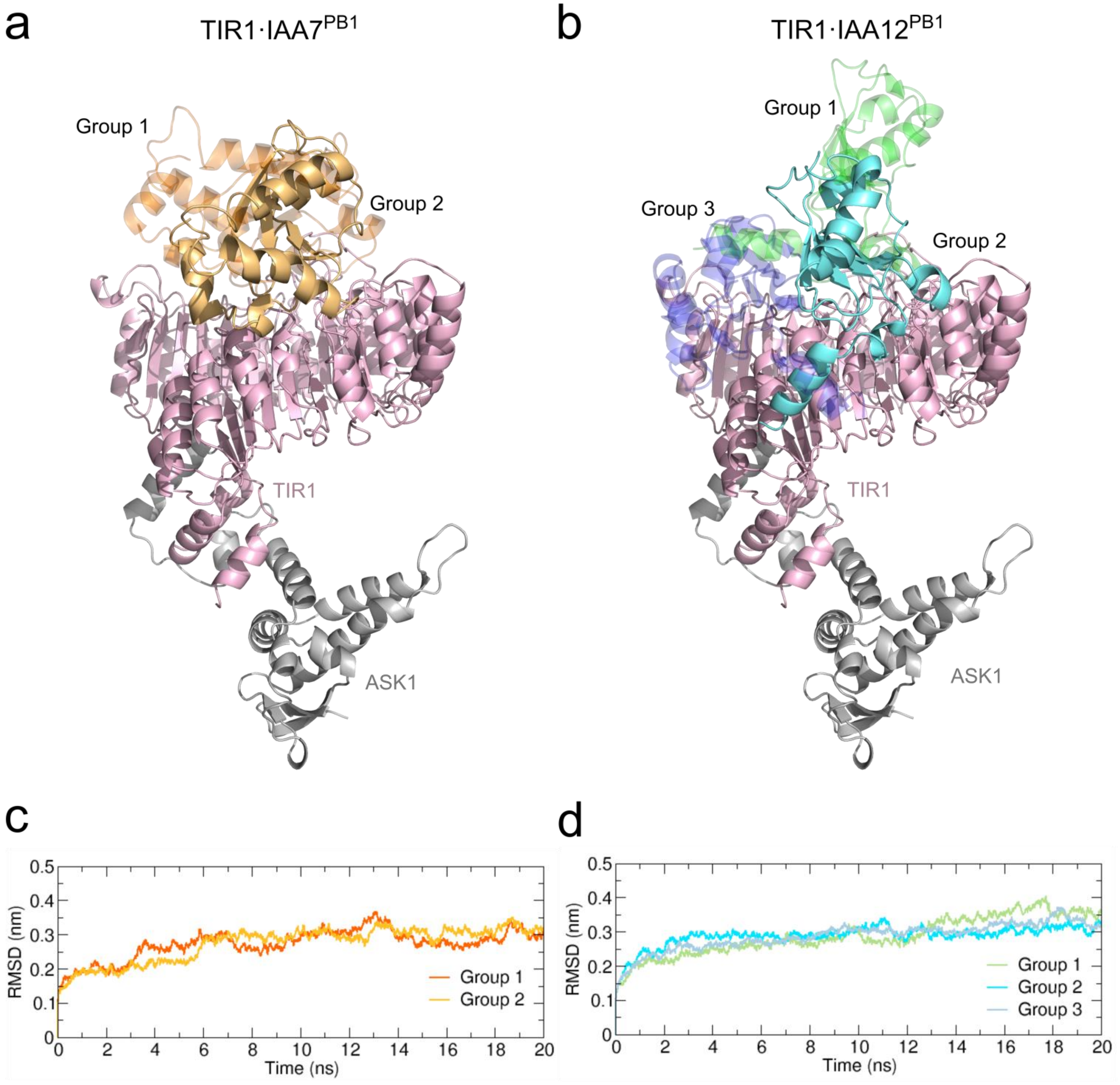

Supplementary Figure 12| HADDOCK docking using crosslinking data restrictions generated stable structural models of TIR1·AUX/IAA^{PB1} complexes. A representative structure for each group of TIR1·IAA7^{PB1} (**a**) (group 1: dark orange, group 2: light orange); and TIR1·IAA12^{PB1} (**b**) (group 1: green, group 2: aquamarine, group 3: dark blue) HADDOCK models are shown. Time evolution (ns) of RMSD values for the backbone atoms of (**c**) TIR1·IAA7^{PB1}and (**d**) TIR1·IAA12^{PB1}models, respect to their initial structure.

Supp. Fig.13

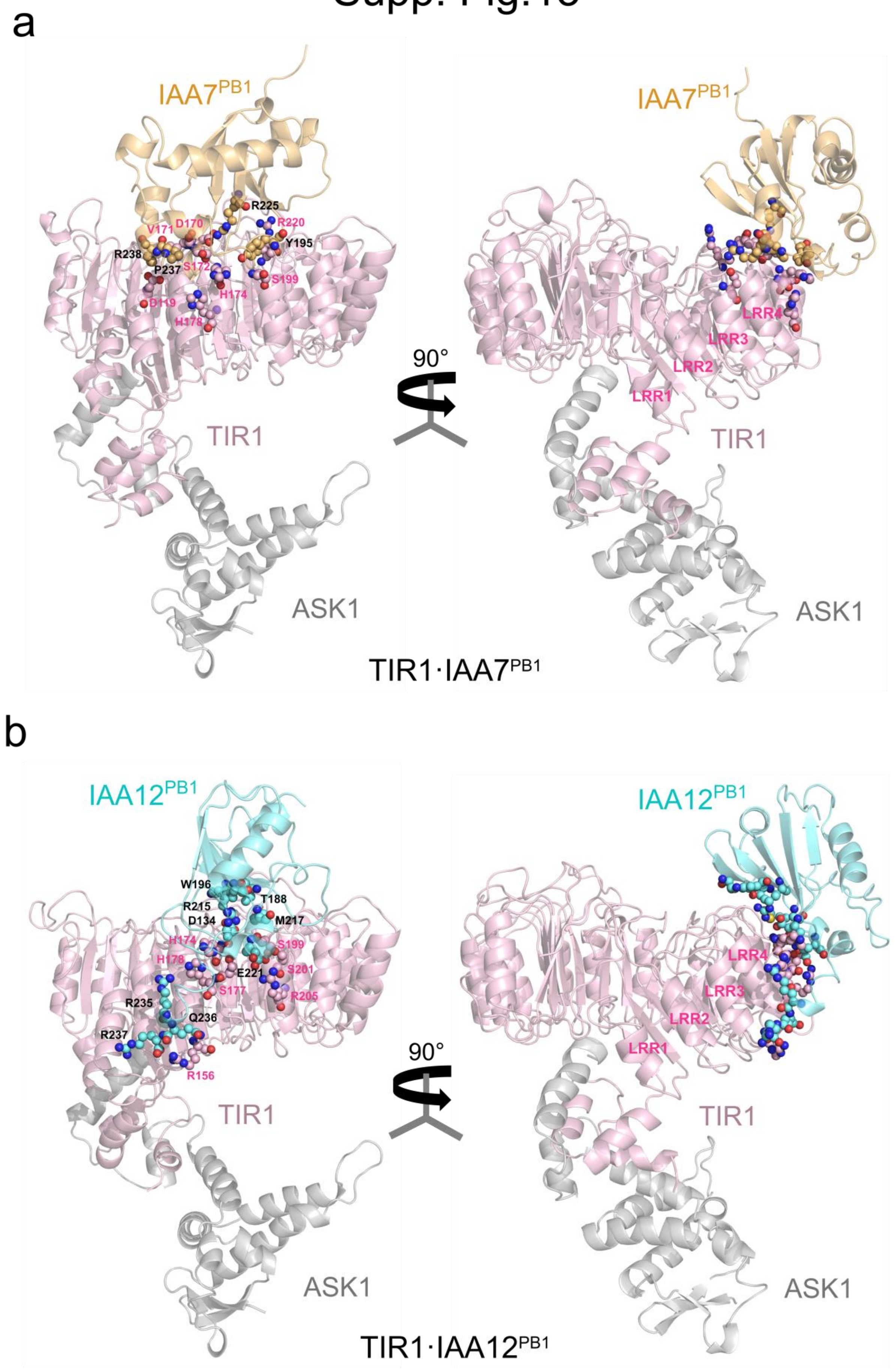

Supplementary Figure 13| Molecular dynamics (MD) simulations revealed the most energetically favorable model from HADDOCK-based docking. (**a-b**) PB1 domains from both IAA7 and IAA12 are positioned over TIR1, interacting with residues from leucine-rich-repeat 3 to 7 (LRR3-7). Energetically relevant residues (small spheres) from TIR1 (light pink), IAA7^{PB1} (light orange), and IAA12^{PB1} (aquamarine) domains for complex stabilization are located in the TIR1·AUX/IAA^{PB1} interface.

Supp. Fig. 14

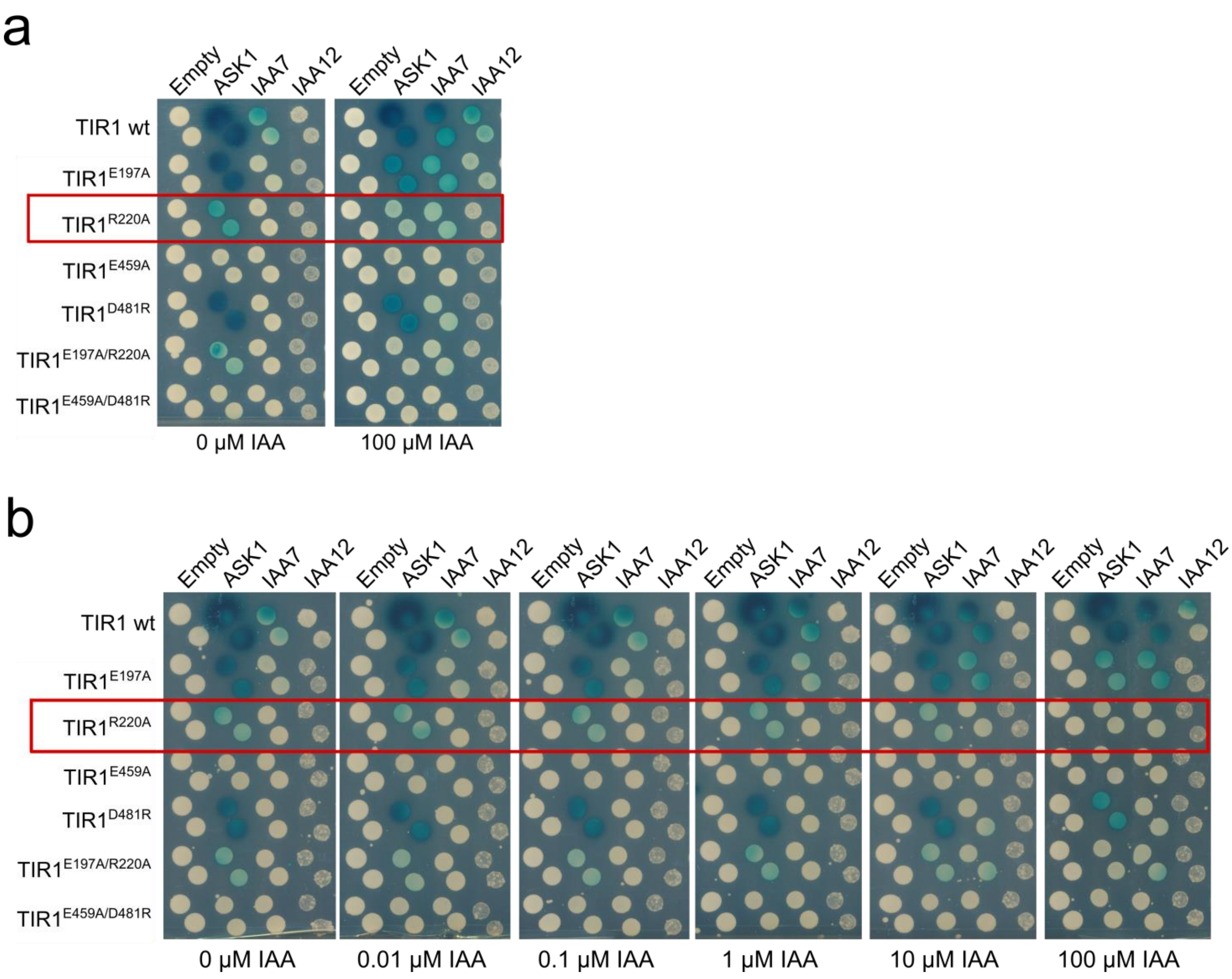

Supplementary Figure 14| Mutation R220A in TIR1 only partially affects TIR1·ASK1 interaction. Initial Y2H screen 1 and 2 (**a-b**, respectively) showing TIR1^{R220A} is only partially impaired in ASK1 recruitment.

Supp. Fig. 15

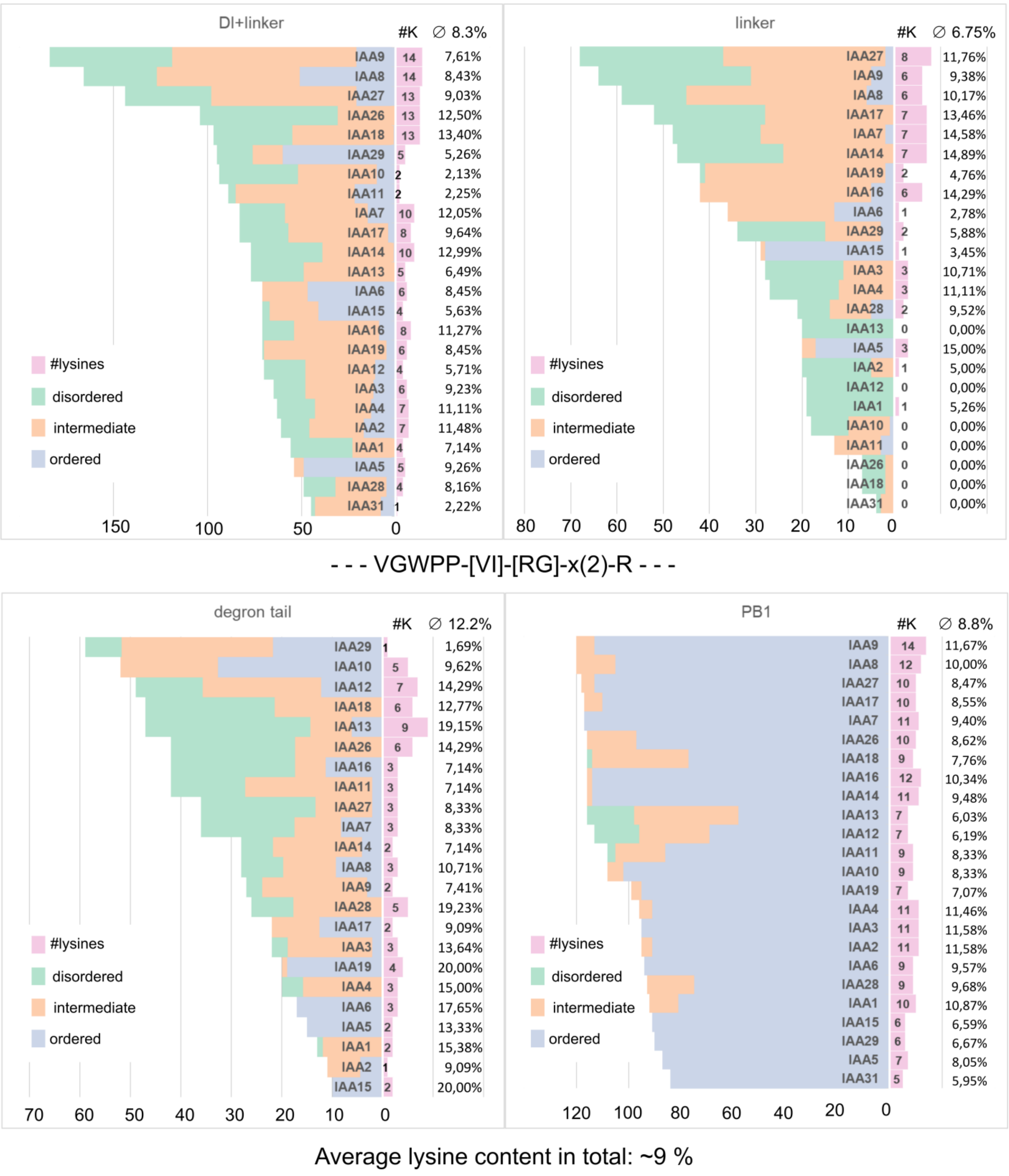

Supplementary Figure 15| Disorder probability and lysine content in different regions of the canonical *At*AUX/IAA proteins. IUPred2A-based prediction for disordered (green), intermediate (orange) and ordered (blue) amino acid residues is shown. Length in AUX/IAA IDRs partially correlate with lysine content and/or disorder. AUX/IAAs with less than 5 lysine residues (pink) in the degron tail show increased lysine content in the PB1 domain (≥10). AUX/IAA degron tails are enriched in ubiquitin acceptors sites (lysine residues, average 12.2% of total residues).

Suppl. Table 1. Restraints used in HADDOCK and DisVis.

Complex	Restraint#	Residue 1	Residue 2	Min. distance (Å)	Max. distance(Å)	Type of restraint
ASK1·TIR1·IAA7	1	TIR1, K226	IAA7, K223	8.0	34.0	Inter XL
	2	TIR1, S229	IAA7, K223	8.0	34.0	Inter XL
	3	IAA7, K94	IAA7, K237	8.0	34.0	Inter XL
	4	IAA7, K94	IAA7, G124	20.0	27.0	Degron tail restraint
ASK1·TIR1·IAA12	1	TIR1, K226	IAA12, K231	8.0	34.0	Inter XL
	2	TIR1, K67	IAA12, K231	8.0	34.0	Inter XL
	3	IAA12, M81	IAA12, P119	24.8	35.0	Degron tail restraint

Supplementary Table 1| Distance restraints used in HADDOCK-based docking and DisVis calculations. Restraints used for generating interaction surfaces with DisVis and/or molecular models with HADDOCK. Defined are the restraints used based on their type and origin.

Suppl. Table 2. Crosslinking-based docking by HADDOCK

Input	# grouped refined structures	group	# structures per group	HADDOCK scores	Buried surface	Van der Waals energy	Electrostatic energy	Restraint violation
ASK1·TIR1·IAA7 without degron tail restraint	175	1	124	-75.7 +/- 4.6	1390.6 +/- 29.4	-37.9 +/- 3.1	-279.1 +/- 15.2	2.5 +/- 0.72
		2	24	-63.8 +/- 10.8	1503.0 +/- 127.2	-47.9 +/- 8.8	-214.5 +/- 95.6	2.7 +/- 1.50
		6	5	-59.4 +/- 10.2	1627.6 +/- 95.8	-46.7 +/- 6.5	-210.5 +/- 15.6	2.3 +/- 1.02
		3	10	-53.6 +/- 9.1	1451.2 +/- 59.6	-32.4 +/- 6.2	-265.6 +/- 60.2	1.6 +/- 0.53
		5	6	-46.7 +/- 7.6	1019.5 +/- 31.2	-38.8 +/- 3.9	-127.3 +/- 32.5	2.5 +/- 0.56
		4	6	-34.2 +/- 7.4	1031.6 +/- 41.0	-22.7 +/- 2.5	-234.4 +/- 27.5	2.1 +/- 0.54
ASK1·TIR1·IAA7 with degron tail restraint	193	2	72	-89.1 +/- 2.5	1689.6 +/- 146.4	-43.5 +/- 3.0	-419.7 +/- 33.1	3.2 +/- 0.84
		1	121	-66.7 +/- 9.3	1395.2 +/- 162.7	-33.5 +/- 3.8	-272.7 +/- 39.0	1.6 +/- 0.36
ASK1·TIR1·IAA12 without degron tail restraint	132 in 13 groups	2	18	-76.3 +/- 10.0	1588.8 +/- 70.0	-38.1 +/- 4.8	-293.4 +/- 45.0	2.5 +/- 0.44
		1	47	-67.1 +/- 12.1	990.6 +/- 15.7	-30.3 +/- 1.6	-260.0 +/- 31.6	1.9 +/- 0.62
		4	9	-66.8 +/- 4.0	1548.8 +/- 140.0	-44.8 +/- 11.7	-257.4 +/- 46.3	2.6 +/- 0.39
		9	5	-66.5 +/- 10.6	1090.0 +/- 38.5	-23.9 +/- 1.4	-381.9 +/- 33.2	3.1 +/- 1.26
		8	5	-61.5 +/- 22.4	1218.9 +/- 164.4	-27.5 +/- 5.4	-404.8 +/- 62.1	2.5 +/- 0.69
		11	5	-51.9 +/- 4.1	1425.8 +/- 80.1	-29.5 +/- 2.0	-357.4 +/- 35.3	2.0 +/- 0.69
		10	5	-45.7 +/- 5.6	1434.8 +/- 70.1	-38.3 +/- 3.4	-230.4 +/- 29.4	1.9 +/- 0.33
		7	6	-41.5 +/- 3.5	1129.3 +/- 38.6	-27.1 +/- 2.5	-267.7 +/- 10.3	2.0 +/- 0.99
		3	10	-35.6 +/- 3.7	1193.6 +/- 31.5	-30.6 +/- 1.7	-89.6 +/- 21.8	2.9 +/- 0.74
		13	4	-33.1 +/- 8.6	1161.6 +/- 120.1	-24.3 +/- 4.5	-207.1 +/- 21.8	1.9 +/- 0.42
ASK1·TIR1·IAA12 with degron tail restraint	196	1	187	-94.2 +/- 9.9	1619.0 +/- 85.6	-35.4 +/- 2.4	-469.4 +/- 48.7	1.8 +/- 0.30
		3	4	-59.8 +/- 14.5	1541.9 +/- 116.0	-40.6 +/- 2.8	-252.6 +/- 58.2	2.8 +/- 0.44
		2	5	-53.9 +/- 10.2	1429.8 +/- 104.0	-38.0 +/- 4.5	-229.1 +/- 35.9	3.0 +/- 1.58

Supplementary Table 2| Resulting clusters from HADDOCK-based docking and their characteristics. Different relevant energetic values from ASK1·TIR1·IAA7 and ASK1·TIR1·IAA12 clusters either in presence or absence of the disorder (degron tail) restraint are shown together with the respective grouping. Results indicate a more defined and more reliable complex identification in the presence of the disorder restraint.

Supp. Table 3. Per-residue energy contributions to the formation of the TIR1·AUX/IAA^{PB1} complexes

Complexes	TIR1 Residues	Conservation within 21 TIR1/AFB-like proteins	prEFED protocol		CAS protocol	
			ΔG_{sc} (kcal/mol) GB^{OBC1}	ΔG_{sc} (kcal/mol) GB^{OBC2}	$\Delta\Delta G$ (kcal/mol) GB^{OBC1}	$\Delta\Delta G$ (kcal/mol) GB^{OBC2}
TIR1·IAA7^{PB1}	D170	5 (+8)	-3.746 +/- 1.11	-5.317 +/- 1.32	-13.678 +/- 2.06	-16.533 +/- 2.36
	R220	9 (+4)	-6.543 +/- 1.35	-6.975 +/- 1.46	-9.585 +/- 2.29	-10.623 +/- 2.54
	D119	19	-2.264 +/- 0.76	-3.364 +/- 0.98	-8.632 +/- 1.48	-10.414 +/- 1.86
	H174	2	-4.902 +/- 0.85	-5.418 +/- 0.97	-7.387 +/- 1.57	-8.255 +/- 1.78
	S172	5	-4.011 +/- 0.62	-4.241 +/- 0.65	-6.671 +/- 1.23	-7.447 +/- 1.28
	S199	2	-3.103 +/- 1.23	-3.375 +/- 1.30	-4.558 +/- 2.04	-5.032 +/- 2.18
	H178	1 (+3)	-2.841 +/- 0.59	-3.041 +/- 0.64	-2.899 +/- 0.95	-3.160 +/- 1.02
	V171	1	-2.536 +/- 0.49	-2.278 +/- 0.51	-2.483 +/- 0.90	-2.099 +/- 0.95
	E197	11 (+4)	2.437 +/- 1.07	2.331 +/- 1.22	-0.149 +/- 2.73	-0.807 +/- 3.14
	D146	8 (+2)	1.988 +/- 0.89	1.959 +/- 0.88	0.709 +/- 1.34	0.385 +/- 1.55
	K226	2	0.685 +/- 0.56	1.077 +/- 0.91	-	-
TIR1·IAA12^{PB1}	R205	6 (+2)	-7.99 +/- 0.90	-7.662 +/- 1.00	-15.253 +/- 1.84	-16.056 +/- 2.03
	R156	19 (+2)	-7.44 +/- 0.97	-7.555 +/- 1.05	-11.491 +/- 1.88	-12.437 +/- 1.99
	H174	2	-4.267 +/- 1.52	-5.195 +/- 1.98	-9.27 +/- 2.46	-10.690 +/- 2.89
	S201	2	-4.058 +/- 0.60	-4.508 +/- 0.64	-7.689 +/- 1.15	-8.963 +/- 1.23
	S199	2	-3.942 +/- 1.15	-4.439 +/- 1.18	-7.448 +/- 2.19	-8.731 +/- 2.36
	H178	1 (+3)	-1.706 +/- 0.93	-2.27 +/- 1.15	-4.349 +/- 1.78	-5.392 +/- 2.09
	S177	11 (+2)	-1.521 +/- 1.32	-1.738 +/- 1.40	-2.833 +/- 2.32	-3.307 +/- 2.54
	K130	2 (+1)	-1.041 +/- 1.27	-0.877 +/- 1.18	-1.745 +/- 2.72	-1.658 +/- 2.82
	S196	5 (+2)	-1.247 +/- 0.41	-1.281 +/- 0.47	-1.286 +/-0.66	-1.423 +/- 0.78
	V171	1	-1.784 +/- 0.38	-1.548 +/- 0.41	-0.767 +/- 0.87	-0.459 +/- 0.94
	A153	5	-1.259 +/- 0.24	-1.286 +/- 0.24	-	-
	D170	5 (+8)	0.459 +/- 0.16	0.059 +/- 0.23	-	-

Supplementary Table 3| Energy contribution of single amino acids to TIR1·AUX/IAA^{PB1} complex formation. Conservation of residues was checked in TIR1/AFB-like proteins in *Arabidopsis thaliana* (uniprot ID: Q570C0, Q9ZR12, Q9LW29, Q9LPW7, A0A178UVM5, A0A178UB83), *Selaginella moellendorffii* (uniprot ID: D8RF91, D8SDE6, D8SG63, D8R5Z3), *Physcomitrella patens* (uniprot ID: A9SYG2, A9TAY1, A9T980, A9SZ50, A9TE08, A9TP16), *Oryza sativa* (uniprot ID: Q0DKP3, Q7XVM8, Q2R3K5, Q8H7P5) and *Marchantia polymorpha* (uniprot ID: A0A2R6WBN4).

Supplementary Methods

Molecular dynamic simulations (MDS) of protein-protein complexes

One refined structure of each group, derived from the cross-link-based docking by HADDOCK incorporating the disorder restraint (2 groups for TIR1·IAA7^{PB1}; 3 groups for TIR1·IAA12^{PB1}), was used as starting structure for MD simulations. The 5 structures were prepared using structure preparation and protonate 3D (pH = 7.5) modules and subsequently minimized with AMBER10 force-field[5] in MOE 2019.0101 (Chemical Computing Group Inc., Montreal, Quebec, Canada).

Molecular dynamic simulations were performed with the GROMACS software package (version 4.6.5)[6]. The parameters corresponding to the proteins were generated with AMBER99SB-ILDN force-field[7] and TIP3P explicit solvation model[8]. Electro-neutrality was guaranteed by adding Na^+ and Cl^- ions into the unit cells at an appropriate ratio to reach a final NaCl concentration of 0.2 mol/L. The protocol employed here to perform MD simulations involves prior energy minimization (EM) and position-restrained equilibration, as outlined by Lindahl [9] for lysozyme in water. The systems were subjected to 50 000 steps of steepest descents minimization with a step size of 0.01 nm[10]. The maximum tolerance was set to 1 000 kJ*mol^{-1}*nm^{-1} and cutoff radii of 1.2 nm were established for the calculation of both van der Waals and short-range electrostatic interactions. The particle mesh Ewald algorithm was used to handle long-range electrostatic interactions[11,12]. The Verlet cutoff-scheme was used, as well as the potential modifier potential-shift-Verlet for both Coulomb and van der Waals interactions. Bond lengths were left unconstrained during EM. Next, the solvent was equilibrated around the system for 50 ps using position restraint dynamics, with force constant of 1,000 kJ*mol^{-1}*nm^{-2} to all the heavy atoms of the proteins. Cutoff radii of 1.2 nm were established for the calculation of van der Waals and electrostatic

interactions. Again, the Verlet cutoff-scheme was used, as well as the potential modifier potential-shift-Verlet for both Coulomb and van der Waals interactions. Newton's equation of motion was solved using the leap-frog integrator[13], with a time step of Δt = 2 fs for a total time of 50 ps (25,000 integration steps). The system was simulated at constant temperature and pressure of 310 K and 1 atm, respectively. In order to accomplish this, we used the Berendsen algorithm[14] for the pressure and Velocity rescaling[15] for the temperature, with time constant (τ) of 3 ps and 0.1 ps, respectively[10]. Bond lengths were constrained by the Linear Constraints Solver algorithm[16]. Random initial velocities were assigned to each atom prior to the MD simulations, obeying the Maxwell-Boltzmann distribution from 50 K to 310 K.[10]

Once the system was equilibrated, we proceeded to the productive dynamic simulation without position restraint[17] for 20 ns. The system simulation was carried out at T = 310 K and p = 1 atm. The Parrinello-Rahman coupling algorithm[18,19] was used to keep pressure constant with a time constant (τ) of 1 ps.[10] The temperature, non-bonded interaction and time step were controlled or set up similarly as in the equilibration run. The snapshots of all runs were saved each 10 ps. Root Mean Square Deviation (RMSD) values for the backbone atoms of TIR1·IAA7^{PB1} and TIR1·IAA12^{PB1} compared to its initial structure, were calculated during the entire simulation using the *g_rms* program (GROMACS v4.6.5)[6].

Effective binding free energy calculations using MM-GBSA

The effective binding free energy (ΔG_{eff}) of the protein-protein complexes formation was calculated using MMPBSA.py from Amber18 package employing the MM-GBSA method[20]. We followed the single trajectory approach, in which the trajectories for the free proteins were extracted from that of the protein-protein complexes. $GB^{OBC}1$ and $GB^{OBC}2$ implicit solvation models were employed[20]. The ΔG_{eff} values were obtained

every 10 ps from the productive MD simulation (20 000 ps). We calculated the cumulative mean (also referred to as accumulated mean) for each of the 2 000 ΔG_{eff} values. We computed the accumulated mean for each position by summing over all previous values and dividing by their number.

Energetically-relevant residues (hot-spots) at the interfaces of TIR1·AUX/IAA PB1 complexes were predicted by using the per-residue effective free energy decomposition (prEFED) protocol implemented in MMPBSA.py[20]. Hot-spot residues were defined as those with a side-chain energy contribution (ΔG_{sc}) of ≤ -1.0 kcal/mol. We used Computational Alanine Scanning (CAS)[20] to further assess per-residue free energy contributions. Alanine single-point mutations were generated on previously identified hot-spots from the prEFED protocol. Both prEFED and CAS protocols were performed from the last 10 ns of the MD simulation.

Supplementary References

1 Uversky, V. N. Natively unfolded proteins: a point where biology waits for physics. *Protein Sci* **11**, 739-756, doi:10.1110/ps.4210102 (2002).

2 Hamdi, K. *et al.* Structural disorder and induced folding within two cereal, ABA stress and ripening (ASR) proteins. *Sci Rep* **7**, 15544, doi:10.1038/s41598-017-15299-4 (2017).

3 Uversky, V. N. What does it mean to be natively unfolded? *Eur J Biochem* **269**, 2-12, doi:10.1046/j.0014-2956.2001.02649.x (2002).

4 Wend, S. *et al.* A quantitative ratiometric sensor for time-resolved analysis of auxin dynamics. *Sci Rep* **3**, 2052, doi:10.1038/srep02052 (2013).

5 AMBER10 (University of California, San Francisco, 2008).

6 Pronk, S. *et al.* GROMACS 4.5: a high-throughput and highly parallel open source molecular simulation toolkit. *Bioinformatics* **29**, 845-854, doi:10.1093/bioinformatics/btt055 (2013).

7 Hornak, V. *et al.* Comparison of multiple Amber force fields and development of improved protein backbone parameters. *Proteins* **65**, 712-725, doi:10.1002/prot.21123 (2006).

8 Jorgensen, W. L., Chandrasekhar, J., Madura, J. D., Impey, R. W. & Klein, M. L. Comparison of Simple Potential Functions for Simulating Liquid Water. *J Chem Phys* **79**, 926-935, doi:Doi 10.1063/1.445869 (1983).

9 Lindahl, E. Molecular dynamics simulations. *Methods Mol Biol* **1215**, 3-26, doi:10.1007/978-1-4939-1465-4_1 (2015).

10 Páll, S., Abraham, M. J., Kutzner, C., Hess, B. & Lindahl, E. in *Solving Software Challenges for Exascale* Vol. 8759 (eds S Markidis & E Laure) 3-27 (2015).

11 Darden, T., York, D. & Pedersen, L. Particle Mesh Ewald: An W log(N) Method for Ewald Sums in Large Systems. *J. Chem. Phys.* **98**, 10089-10093 (1993).

12 Essmann, U. *et al.* A Smooth Particle Mesh Ewald Method. *J. Chem. Phys.* **103**, 8577-8592 (1995).

13 Verlet, L. Computer "Experiments" on Classical Fluids. I. Thermodynamical Properties of Lennard-Jones Molecules. *Phys. Rev.* **159**, 98-103 (1967).

14 Berendsen, H. J. C., Postma, J. P. M., DiNola, A. & Haak, J. R. Molecular Dynamics with Coupling to an External Bath. . *J. Chem. Phys.* **81** (1984).

15 Bussi, G., Donadio, D. & Parrinello, M. Canonical sampling through velocity rescaling. *J. Chem. Phys.* **126** (2007).

16 Hess, B. & et al. A linear constraint solver for molecular simulations. *J. Comput. Chem.* **18**, 1463–1472 (1997).

17 Schneider, T. & Stoll, E. Molecular-Dynamics study of a 3-dimensional one-component model for distortive phase-transitions. *Phys. Rev. B* **17**, 1302-1322 (1978).

18 Parrinello, M. & Rahman, A. Polymorphic Transitions in Single Crystals: A New Molecular Dynamics Method. *J. Appl. Phys.* **52**, 7182-7190 (1981).

19 Nosé, S. & Klein, M. L. Constant pressure molecular dynamics for molecular systems. *Mol. Phys.* **50**, 1055-1076 (1983).

20 AMBER 2018 (University of California, San Francisco, 2018).
