# Supplementary material for: Flexibility of intrinsically disordered degrons in AUX/IAA proteins reinforces auxin co-receptor assemblies
Source: Nat Commun. 2020 May 8;11:2277. doi: 10.1038/s41467-020-16147-2 (PMC7210949; doi:10.1038/s41467-020-16147-2)
Supplement: Supplementary file 3 — Description of Additional Supplementary Files [file 41467_2020_16147_MOESM3_ESM.docx]

**Description of Additional Supplementary Files**

**File name:** Supplementary Data 1

**Description:** Supplementary Data 1 contains two excel sheets: One with all Primer sequences used to generate the constructs mentioned in the manuscript and another one containing all used Aux/IAA amino acid sequences with their Uniprot ID and a hyperlink to the Uniprot website of each.

**File name:** Supplementary Data 2

**Description:** LC/MS2 based identification of ubiquitin-modified peptides of IAA7 or IAA12 from in vitro ubiquitylation reactions carried out and presented in Figure 3.

**File name:** Supplementary Data 3

**Description:** Results of the crosslinking analysis via LC/MS2 for IAA7 ± TIR1 for all replica and in the absence and presence of auxin.

**File name:** Supplementary Data 4

**Description:** Results of the crosslinking analysis via LC/MS2 for IAA12 ± TIR1 for all replica and in the absence and presence of auxin.

**File name:** Supplementary Data 5

**Description:** Supplementary Dataset 05 contains all starting material and parameter files needed for modeller-based homology modelling.

**File name:** Supplementary Data 6

**Description:** Supplementary Dataset 06 contains all starting material and parameter files needed for haddock-based molecular docking of TIR1∙IAA7 PB1 and TIR1∙IAA12<sup>PB1</sup> complexes.

**File name:** Supplementary Data 7

**Description:** Supplementary Dataset 07 contains all starting material and parameter files needed for DisVis analysis of TIR1∙IAA7 PB1 and TIR1∙IAA12 PB1 complexes.

**Name:** Supplementary Data 8

**Description:** Model for ASK1∙TIR1∙AUX/IAA complex assembly fine-tuned by IDRs flanking the AUX/IAA degron. The F-Box Protein TIR1 of the SCF<sup>TIR1</sup> E3 ubiquitin ligase recruits AUX/IAA targets for their ubiquitylation and degradation. The phytohormone auxin and a core degron in AUX/IAAs are essential for AUX/IAA recognition. Intrinsically disordered regions (IDRs) flanking the degron provide high flexibility and an extended fold to AUX/IAAs, influencing TIR1∙AUX/IAA complex formation. At least two different routes are possible for dynamic AUX/IAA recruitment and UPS-mediated degradation: i auxin-triggered association between TIR1 and the AUX/IAA degron paves the way for positioning adjacent IDRs, which exposes ubiquitin acceptor sites for efficient ubiquitylation; ii transient auxin-independent interactions between IDRs, as well as the PB1 domain in AUX/IAAs, and two patches (clusters 1 and 2) of residues at opposite sides of TIR1, assist on auxin binding and offer tailored positioning. Residues R220 and S201 from cluster 1 (right zoom in) and D481 from cluster 2 (left zoom in) in TIR1 play a major role in TIR1∙AUX/IAA complex formation. The residency time of an AUX/IAA target on TIR1, enables processivity of AUX/IAA ubiquitylation, and impinges on availability of IDRs as initiation sites for degradation by the 26S proteasome.
