# Supplementary material for: Flexibility of intrinsically disordered degrons in AUX/IAA proteins reinforces auxin co-receptor assemblies
Source: Nat Commun. 2020 May 8;11:2277. doi: 10.1038/s41467-020-16147-2 (PMC7210949; doi:10.1038/s41467-020-16147-2)
Supplement: Supplementary file 9 — Supplementary Data 6 [file 41467_2020_16147_MOESM9_ESM.gz › haddock_files/IAA12/With_disorder_restraint/05_multi-IAA12_COM2.docx]

**HADDOCK server status for docking run /4156569441/05_multi-IAA12_COM2**

**Status: FINISHED**

Your HADDOCK run has successfully completed. The complete run can be downloaded as a gzipped tar file [**here**](http://milou.science.uu.nl/serviceresults/HADDOCK2.2/4156569441/05_multi-IAA12_COM2.tgz) (Note that there might be a delay in the generation of this archive - in case of errors try again). The file containing your docking parameters is [**here**](https://milou.science.uu.nl/serviceresults/HADDOCK2.2/4156569441/05_multi-IAA12_COM2/haddockparam.web).

Please cite the following papers in your work: 
G.C.P van Zundert, J.P.G.L.M. Rodrigues, M. Trellet, C. Schmitz, P.L. Kastritis, E. Karaca, A.S.J. Melquiond, M. van Dijk, S.J. de Vries and A.M.J.J. Bonvin (2016). "[The HADDOCK2.2 webserver: User-friendly integrative modeling of biomolecular complexes](http://dx.doi.org/doi:10.1016/j.jmb.2015.09.014)."
*J. Mol. Biol.*, **428**, 720-725 (2015).

- Wassenaar *et al.*, [WeNMR: Structural Biology on the Grid.](http://link.springer.com/article/10.1007/s10723-012-9246-z)
  *J. Grid. Comp.*, **10**, 743-767 (2012).

And for the use of the WeNMR Grid resources please please add the following acknowledgement:
*"The FP7 [WeNMR](http://www.wenmr.eu/) (project# 261572), H2020*[*West-Life*](http://www.west-life.eu/)*(project# 675858) and the*[*EOSC-hub*](http://eosc-hub.eu/)*(project# 777536) European e-Infrastructure projects are acknowledged for the use of their web portals, which make use of the*[*EGI*](http://www.egi.eu/)*infrastructure with the dedicated support of CESNET-MetaCloud, INFN-PADOVA, NCG-INGRID-PT, TW-NCHC, SURFsara and NIKHEF, and the additional support of the national GRID Initiatives of Belgium, France, Italy, Germany, the Netherlands, Poland, Portugal, Spain, UK, Taiwan and the US Open Science Grid."*

**How would you rate your experience with our portal?**sentiment_very_dissatisfied sentiment_dissatisfied sentiment_neutral sentiment_satisfied sentiment_very_satisfied

**Questions / feedback ?**[ask.bioexcel.eu](http://ask.bioexcel.eu/)

**Interested in interaction prediction?** Consider attending the 7th CAPRI evaluation meeting on April 3-5, 2019 in Hinxton, UK. More info [**here**](http://www.capri-docking.org/events/). The meeting will be preceeded by a two days hands-on workshop sponsored by INSTRUCT-ERIC on integrative modeling of protein-protein interactions and larger multi-molecular assemblies.

**Summary**

HADDOCK clustered**196**structures in**3**cluster(s), which represents**98.0 %**of the water-refined models HADDOCK generated. Note that currently the maximum number of models considered for clustering is 200.

The statistics of the top 10 clusters are shown below. The top cluster is the most reliable according to HADDOCK. Its Z-score indicates how many standard deviations from the average this cluster is located in terms of score (the more negative the better).

A [graphical representation](https://milou.science.uu.nl/serviceresults/HADDOCK2.2/4156569441/05_multi-IAA12_COM2/#graphics) of the results is also provided at the bottom of the page.

Cluster 1

| HADDOCK score | -94.2 +/- 9.9 |
| --- | --- |
| Cluster size | 187 |
| RMSD from the overall lowest-energy structure | 0.5 +/- 0.3 |
| Van der Waals energy | -35.4 +/- 2.4 |
| Electrostatic energy | -469.4 +/- 48.7 |
| Desolvation energy | 34.9 +/- 15.3 |
| Restraints violation energy | 1.8 +/- 0.30 |
| Buried Surface Area | 1619.0 +/- 85.6 |
| Z-Score | -1.4 |

| Nr 1 best structure | [Download structure](https://milou.science.uu.nl/serviceresults/HADDOCK2.2/4156569441/05_multi-IAA12_COM2/cluster1_1.pdb) | [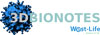](javascript:void(0)) |
| --- | --- | --- |
| Nr 2 best structure | [Download structure](https://milou.science.uu.nl/serviceresults/HADDOCK2.2/4156569441/05_multi-IAA12_COM2/cluster1_2.pdb) |  |
| Nr 3 best structure | [Download structure](https://milou.science.uu.nl/serviceresults/HADDOCK2.2/4156569441/05_multi-IAA12_COM2/cluster1_3.pdb) |  |
| Nr 4 best structure | [Download structure](https://milou.science.uu.nl/serviceresults/HADDOCK2.2/4156569441/05_multi-IAA12_COM2/cluster1_4.pdb) |  |

Cluster 3

| HADDOCK score | -59.8 +/- 14.5 |
| --- | --- |
| Cluster size | 4 |
| RMSD from the overall lowest-energy structure | 11.8 +/- 0.1 |
| Van der Waals energy | -40.6 +/- 2.8 |
| Electrostatic energy | -252.6 +/- 58.2 |
| Desolvation energy | 31.1 +/- 17.6 |
| Restraints violation energy | 2.8 +/- 0.44 |
| Buried Surface Area | 1541.9 +/- 116.0 |
| Z-Score | 0.5 |

| Nr 1 best structure | [Download structure](https://milou.science.uu.nl/serviceresults/HADDOCK2.2/4156569441/05_multi-IAA12_COM2/cluster3_1.pdb) | [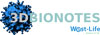](javascript:void(0)) |
| --- | --- | --- |
| Nr 2 best structure | [Download structure](https://milou.science.uu.nl/serviceresults/HADDOCK2.2/4156569441/05_multi-IAA12_COM2/cluster3_2.pdb) |  |
| Nr 3 best structure | [Download structure](https://milou.science.uu.nl/serviceresults/HADDOCK2.2/4156569441/05_multi-IAA12_COM2/cluster3_3.pdb) |  |
| Nr 4 best structure | [Download structure](https://milou.science.uu.nl/serviceresults/HADDOCK2.2/4156569441/05_multi-IAA12_COM2/cluster3_4.pdb) |  |

Cluster 2

| HADDOCK score | -53.9 +/- 10.2 |
| --- | --- |
| Cluster size | 5 |
| RMSD from the overall lowest-energy structure | 7.4 +/- 0.1 |
| Van der Waals energy | -38.0 +/- 4.5 |
| Electrostatic energy | -229.1 +/- 35.9 |
| Desolvation energy | 29.6 +/- 5.4 |
| Restraints violation energy | 3.0 +/- 1.58 |
| Buried Surface Area | 1429.8 +/- 104.0 |
| Z-Score | 0.9 |

| Nr 1 best structure | [Download structure](https://milou.science.uu.nl/serviceresults/HADDOCK2.2/4156569441/05_multi-IAA12_COM2/cluster2_1.pdb) | [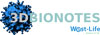](javascript:void(0)) |
| --- | --- | --- |
| Nr 2 best structure | [Download structure](https://milou.science.uu.nl/serviceresults/HADDOCK2.2/4156569441/05_multi-IAA12_COM2/cluster2_2.pdb) |  |
| Nr 3 best structure | [Download structure](https://milou.science.uu.nl/serviceresults/HADDOCK2.2/4156569441/05_multi-IAA12_COM2/cluster2_3.pdb) |  |
| Nr 4 best structure | [Download structure](https://milou.science.uu.nl/serviceresults/HADDOCK2.2/4156569441/05_multi-IAA12_COM2/cluster2_4.pdb) |  |

Results analysis

The results and graphics presented below are based on water-refined models generated by HADDOCK. The clusters (indicated in color in the graphs) are calculated based on the interface-ligand RMSDs calculated by HADDOCK, with the interface defined automatically based on all observed contacts. The various structural analysis [(FCC, i-RMSD and l-RMSD)](https://milou.science.uu.nl/serviceresults/HADDOCK2.2/4156569441/05_multi-IAA12_COM2/#criteria) are made with respect to the best HADDOCK model (the one with the lowest HADDOCK score).

| [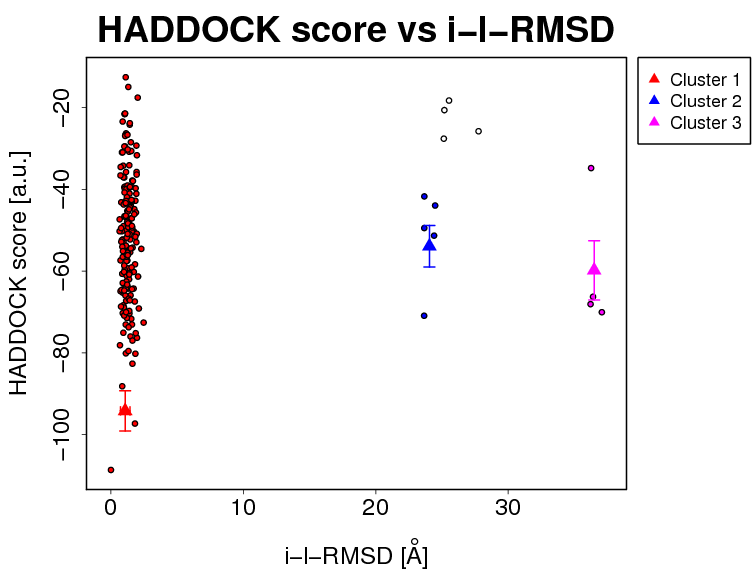](https://milou.science.uu.nl/serviceresults/HADDOCK2.2/4156569441/05_multi-IAA12_COM2/ilrmsd_graph.png) | |
| --- | --- |
| [[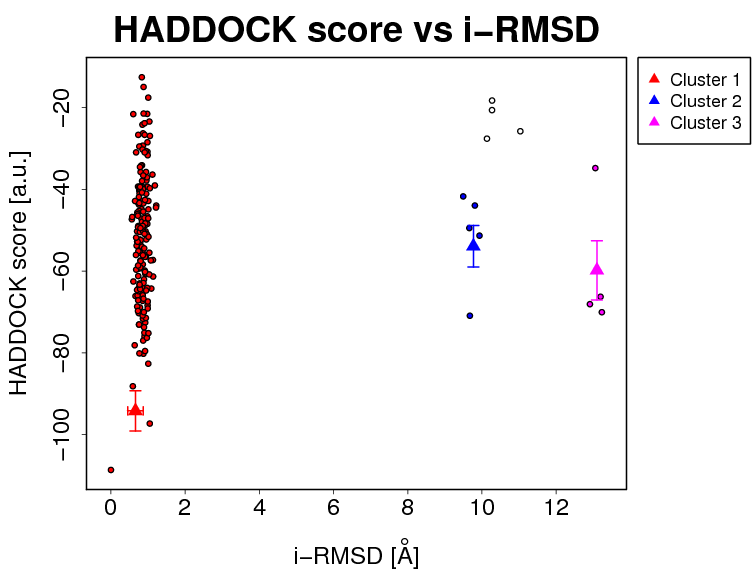](https://milou.science.uu.nl/serviceresults/HADDOCK2.2/4156569441/05_multi-IAA12_COM2/irmsd_graph.png)](https://milou.science.uu.nl/serviceresults/HADDOCK2.2/4156569441/05_multi-IAA12_COM2/irmsd_graph.png) | [[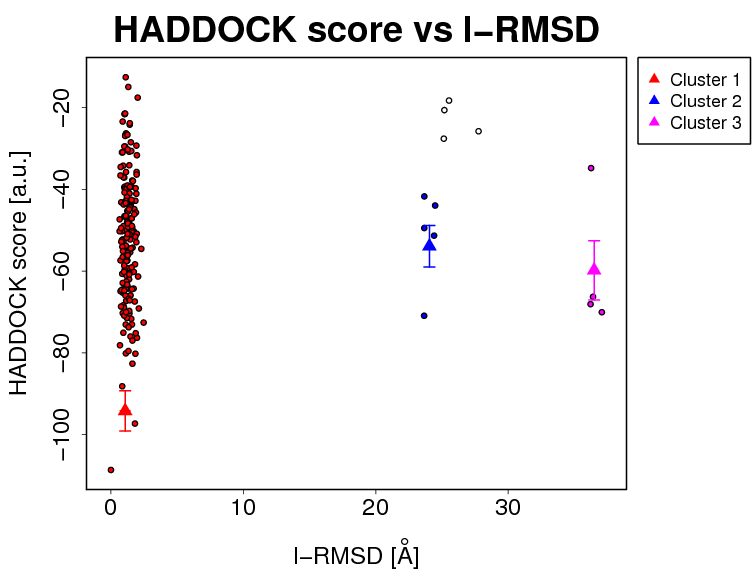](https://milou.science.uu.nl/serviceresults/HADDOCK2.2/4156569441/05_multi-IAA12_COM2/lrmsd_graph.png)](https://milou.science.uu.nl/serviceresults/HADDOCK2.2/4156569441/05_multi-IAA12_COM2/lrmsd_graph.png) |
| [[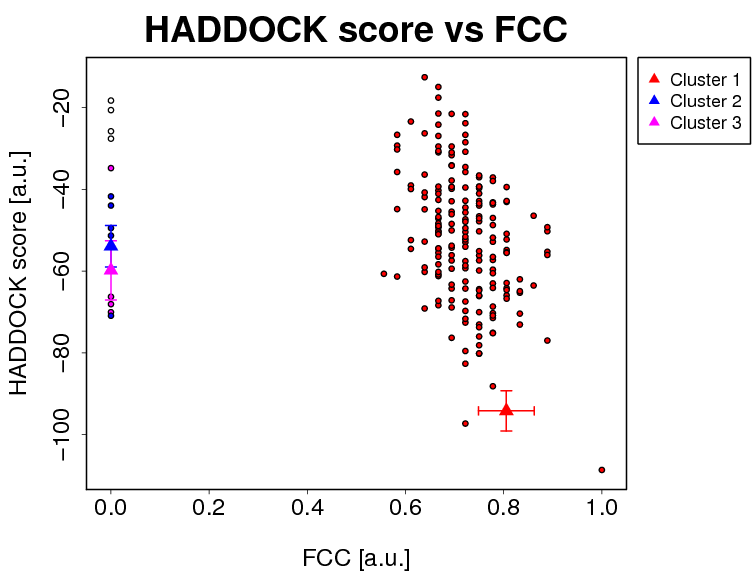](https://milou.science.uu.nl/serviceresults/HADDOCK2.2/4156569441/05_multi-IAA12_COM2/fnat_graph.png)](https://milou.science.uu.nl/serviceresults/HADDOCK2.2/4156569441/05_multi-IAA12_COM2/fnat_graph.png) | [[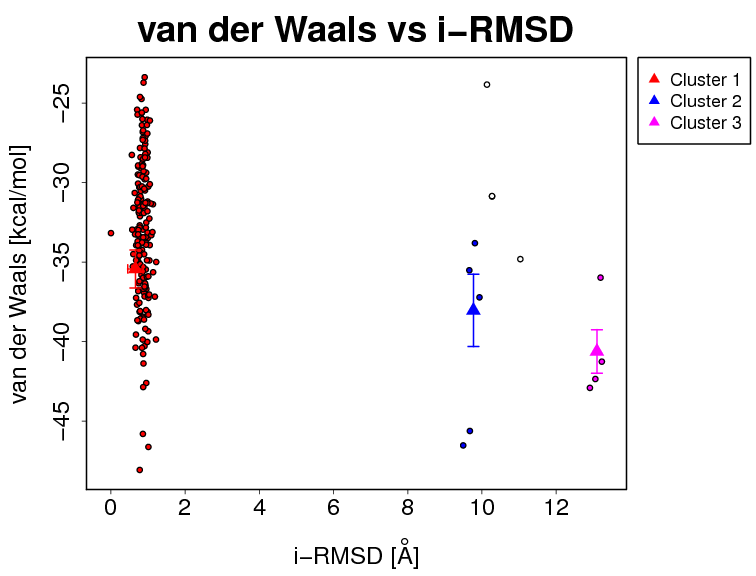](https://milou.science.uu.nl/serviceresults/HADDOCK2.2/4156569441/05_multi-IAA12_COM2/vdw_graph.png)](https://milou.science.uu.nl/serviceresults/HADDOCK2.2/4156569441/05_multi-IAA12_COM2/vdw_graph.png) |
| [[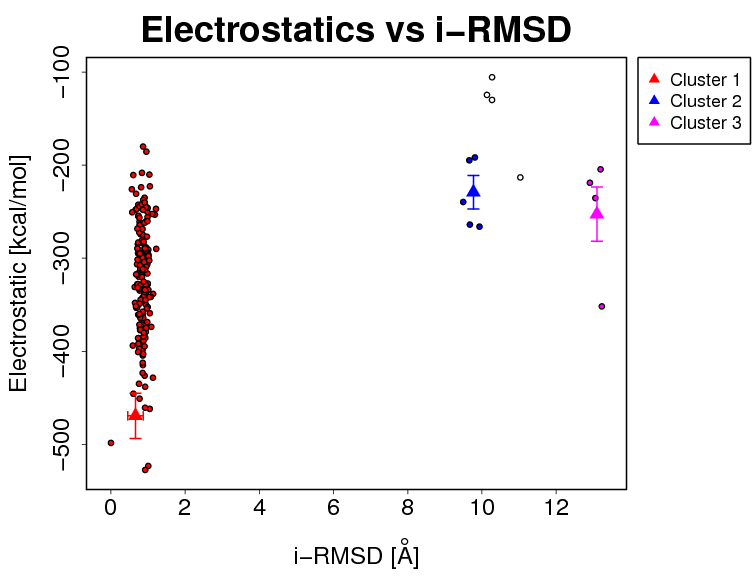](https://milou.science.uu.nl/serviceresults/HADDOCK2.2/4156569441/05_multi-IAA12_COM2/elec_graph.png)](https://milou.science.uu.nl/serviceresults/HADDOCK2.2/4156569441/05_multi-IAA12_COM2/elec_graph.png) | [[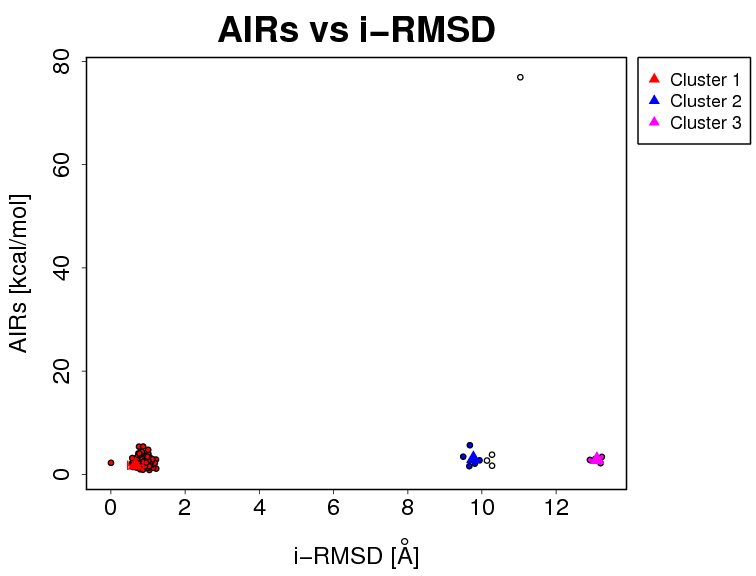](https://milou.science.uu.nl/serviceresults/HADDOCK2.2/4156569441/05_multi-IAA12_COM2/air_graph.png)](https://milou.science.uu.nl/serviceresults/HADDOCK2.2/4156569441/05_multi-IAA12_COM2/air_graph.png) |

Supplementary information:

**i-RMSD** -> interface-RMSD calculated on the backbone (CA,C,N,O,P) atoms of all residues involved in intermolecular contact using a 10Å cutoff 
**l-RMSD** -> ligand-RMSD calculated on the backbone atoms (CA,C,N,O,P) of all (N>1) molecules after fitting on the backbone atoms of the first (N=1) molecule 
**FCC** -> Fraction of common contacts. The intermolecular contacts are defined based on the best HADDOCK model using a 5Å cutoff (see [Rodrigues et al, Proteins 2012](http://onlinelibrary.wiley.com/doi/10.1002/prot.24078/abstract))
**a.u.** -> Arbitrary Units 
The cluster averages and standard deviations are indicated by colored dots with associated error bars. The average values are calculated on the best 4 structures of each clusters (based on the HADDOCK score).

**Note that HADDOCK results are deleted after one week.**
