# Supplementary material for: Flexibility of intrinsically disordered degrons in AUX/IAA proteins reinforces auxin co-receptor assemblies
Source: Nat Commun. 2020 May 8;11:2277. doi: 10.1038/s41467-020-16147-2 (PMC7210949; doi:10.1038/s41467-020-16147-2)
Supplement: Supplementary file 10 — Supplementary Data 7 [file 41467_2020_16147_MOESM10_ESM.gz › DisVis_only_files/DISVIS_IAA12/noDIs-restraints/results_eWU_3xA3Xv47/results.html]

DisVis - Run eWU\_3xA3Xv47


- Home >>
- DISVIS >>
- **index**

DISVIS

@BonvinLab

- HADDOCK
- CPORT
- DISVIS
- POWERFIT
- PRODIGY
- SPOTON
- 3D-DART
- BONVIN LAB

- About
- Submit
- Register
- Examples
- Help/Manual
- Tutorial
- Support Forum

- Welcome to the
  local
  DISVIS webserver! >>

:   **Run IAA12\_res\_all-nodis (eWU\_3xA3Xv47)**
:   **Status: FINISHED**
:   Your DisVis run has successfully completed.
:   Please cite the following papers in your work:
:   G.C.P. van Zundert, M. Trellet, J. Schaarschmidt, Z. Kurkcuoglu, M. David, M. Verlato, A. Rosato and A.M.J.J. Bonvin.
:   and add the following acknowledgment:
:   The FP7 WeNMR (project# 261572), H2020 West-Life
    (project# 675858) and the EOSC-hub (project# 777536) European e-Infrastructure projects
    are acknowledged for the use of their web portals, which make use of the EGI
    infrastructure with the dedicated support of CESNET-MetaCloud, INFN-PADOVA, NCG-INGRID-PT, TW-NCHC, SURFsara and NIKHEF,
    and the additional support of the national GRID Initiatives of Belgium, France, Italy, Germany, the Netherlands, Poland,
    Portugal, Spain, UK, Taiwan and the US Open Science Grid.
:   **Note:** Your results will be stored for 14 days before being removed from the server, please make a backup as soon as possible.
:   **How would you rate your experience with our portal?** 
    *sentiment\_very\_dissatisfied*
    *sentiment\_dissatisfied*
    *sentiment\_neutral*
    *sentiment\_satisfied*
    *sentiment\_very\_satisfied*
     Thank you!*done*
:   **Questions / feedback ?**  ask.bioexcel.eu
:   **Please also consider giving us some feedback by filling our online survey.**

Accessible Complexes
:   The table below lists the number of complexes consistent with at least N restraints as raw number
    and as fraction of all complexes.
:   | Number of consistent restraints (N) | Number of accessible complexes consistent with at least N restraints | Fraction of accessible complexes consistent with at least N restraints |
    | --- | --- | --- |
    | 0 | 43092045 | 100.00 % |
    | 1 | 7484769 | 17.37 % |
    | 2 | 277982 | 0.65 % |

z-Score
:   **IMPORTANT!**  Complexes consistent with all restraints you provided exist indicating that
    there are no false positives among your restraints. The tables listing z-Scores and Violations can thus be
    ignored but are provided as part of the DisVis output nevertheless.
:   | # | Restraint | Average violated fraction | Standard deviation | Z-score |
    | --- | --- | --- | --- | --- |
    | 1 | A226(CA)-B233(CA) | 0.22 | 0.22 | -1.00 |
    | 2 | A67(CA)-B233(CA) | 0.26 | 0.26 | 1.00 |

Violations
:   **IMPORTANT!**  Complexes consistent with all restraints you provided exist indicating that
    there are no false positives among your restraints. The tables listing z-Scores and Violations can thus be
    ignored but are provided as part of the DisVis output nevertheless.
:   | Number of consistent restraints (N) | Restraint 1 | Restraint 2 |
    | --- | --- | --- |
    | 1 | 0.44 | 0.52 |

- HADDOCK
- CPORT
- DISVIS
- POWERFIT
- PRODIGY
- SPOTON
- 3D-DART
- BONVIN LAB

2008-2017 © Computational Structural Biology group / Science Faculty / Utrecht University. All rights reserved.
