# Supplementary material for: Flexibility of intrinsically disordered degrons in AUX/IAA proteins reinforces auxin co-receptor assemblies
Source: Nat Commun. 2020 May 8;11:2277. doi: 10.1038/s41467-020-16147-2 (PMC7210949; doi:10.1038/s41467-020-16147-2)
Supplement: Supplementary file 11 — Supplementary Data 8 [file 41467_2020_16147_MOESM11_ESM.gz › disvis_haddock_files/IAA07/Summary.docx]

**HADDOCK server status for docking run /4156569441/divis_haddock_iaa07_2**

**Status: FINISHED**

Your HADDOCK run has successfully completed. The complete run can be downloaded as a gzipped tar file [**here**](http://milou.science.uu.nl/serviceresults/HADDOCK2.2/4156569441/divis_haddock_iaa07_2.tgz) (Note that there might be a delay in the generation of this archive - in case of errors try again). The file containing your docking parameters is [**here**](file:///W:\05_FG_Signalintegration\Michael_Niemeyer\Results\Modeling\HADDOCK%20crosslinking-based%20models\divis_haddock_iaa07_2\divis_haddock_iaa07_2\haddockparam.web).

Please cite the following papers in your work:

- G.C.P van Zundert, J.P.G.L.M. Rodrigues, M. Trellet, C. Schmitz, P.L. Kastritis, E. Karaca, A.S.J. Melquiond, M. van Dijk, S.J. de Vries and A.M.J.J. Bonvin (2016). "[The HADDOCK2.2 webserver: User-friendly integrative modeling of biomolecular complexes](http://dx.doi.org/doi:10.1016/j.jmb.2015.09.014)."
  *J. Mol. Biol.*, **428**, 720-725 (2015).
- Wassenaar *et al.*, [WeNMR: Structural Biology on the Grid.](http://link.springer.com/article/10.1007/s10723-012-9246-z)
  *J. Grid. Comp.*, **10**, 743-767 (2012).

And for the use of the WeNMR Grid resources please please add the following acknowledgement:
*"The FP7 [WeNMR](http://www.wenmr.eu) (project# 261572), H2020*[*West-Life*](http://www.west-life.eu)*(project# 675858) and the*[*EOSC-hub*](http://eosc-hub.eu)*(project# 777536) European e-Infrastructure projects are acknowledged for the use of their web portals, which make use of the*[*EGI*](http://www.egi.eu)*infrastructure with the dedicated support of CESNET-MCC, INFN-PADOVA, NCG-INGRID-PT, TW-NCHC, SURFsara and NIKHEF, and the additional support of the national GRID Initiatives of Belgium, France, Italy, Germany, the Netherlands, Poland, Portugal, Spain, UK, Taiwan and the US Open Science Grid."*

**How would you rate your experience with our portal?**sentiment_very_dissatisfied sentiment_dissatisfied sentiment_neutral sentiment_satisfied sentiment_very_satisfied

**Questions / feedback ?**[ask.bioexcel.eu](http://ask.bioexcel.eu)

**Announcing the 2020**[**EMBO practical course**](http://meetings.embo.org/event/20-biomolecular-interactions)**on Integrative Modelling of Biomolecular Interactions**.
It will take place at the Izmir Biomedicine and Genome Center (IBG), May 10-15, 2020.

**Summary**

HADDOCK clustered**179**structures in**9**cluster(s), which represents**89.5 %**of the water-refined models HADDOCK generated. Note that currently the maximum number of models considered for clustering is 200.

**WARNING**: Clustering with default parameters did not produce any cluster, cluster minimum size went from **4** to
The statistics of the top 10 clusters are shown below. The top cluster is the most reliable according to HADDOCK. Its Z-score indicates how many standard deviations from the average this cluster is located in terms of score (the more negative the better). A [graphical representation](file:///W:\05_FG_Signalintegration\Michael_Niemeyer\Results\Modeling\HADDOCK%20crosslinking-based%20models\divis_haddock_iaa07_2\divis_haddock_iaa07_2\index.html#graphics) of the results is also provided at the bottom of the page.

Cluster 6

| HADDOCK score | -80.0 +/- 13.6 |
| --- | --- |
| Cluster size | 12 |
| RMSD from the overall lowest-energy structure | 1.0 +/- 0.7 |
| Van der Waals energy | -68.0 +/- 2.4 |
| Electrostatic energy | -428.7 +/- 19.1 |
| Desolvation energy | 47.3 +/- 13.7 |
| Restraints violation energy | 264.4 +/- 66.76 |
| Buried Surface Area | 2527.3 +/- 108.6 |
| Z-Score | -1.1 |

| Nr 1 best structure | [Download structure](file:///W:\05_FG_Signalintegration\Michael_Niemeyer\Results\Modeling\HADDOCK%20crosslinking-based%20models\divis_haddock_iaa07_2\divis_haddock_iaa07_2\cluster6_1.pdb) | [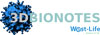](javascript:void(0)) |
| --- | --- | --- |
| Nr 2 best structure | [Download structure](file:///W:\05_FG_Signalintegration\Michael_Niemeyer\Results\Modeling\HADDOCK%20crosslinking-based%20models\divis_haddock_iaa07_2\divis_haddock_iaa07_2\cluster6_2.pdb) |  |
| Nr 3 best structure | [Download structure](file:///W:\05_FG_Signalintegration\Michael_Niemeyer\Results\Modeling\HADDOCK%20crosslinking-based%20models\divis_haddock_iaa07_2\divis_haddock_iaa07_2\cluster6_3.pdb) |  |
| Nr 4 best structure | [Download structure](file:///W:\05_FG_Signalintegration\Michael_Niemeyer\Results\Modeling\HADDOCK%20crosslinking-based%20models\divis_haddock_iaa07_2\divis_haddock_iaa07_2\cluster6_4.pdb) |  |

Cluster 1

| HADDOCK score | -80.0 +/- 3.2 |
| --- | --- |
| Cluster size | 45 |
| RMSD from the overall lowest-energy structure | 10.3 +/- 0.0 |
| Van der Waals energy | -77.5 +/- 3.2 |
| Electrostatic energy | -381.7 +/- 42.3 |
| Desolvation energy | 31.9 +/- 6.8 |
| Restraints violation energy | 419.5 +/- 72.72 |
| Buried Surface Area | 2534.4 +/- 38.0 |
| Z-Score | -1.1 |

| Nr 1 best structure | [Download structure](file:///W:\05_FG_Signalintegration\Michael_Niemeyer\Results\Modeling\HADDOCK%20crosslinking-based%20models\divis_haddock_iaa07_2\divis_haddock_iaa07_2\cluster1_1.pdb) | [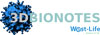](javascript:void(0)) |
| --- | --- | --- |
| Nr 2 best structure | [Download structure](file:///W:\05_FG_Signalintegration\Michael_Niemeyer\Results\Modeling\HADDOCK%20crosslinking-based%20models\divis_haddock_iaa07_2\divis_haddock_iaa07_2\cluster1_2.pdb) |  |
| Nr 3 best structure | [Download structure](file:///W:\05_FG_Signalintegration\Michael_Niemeyer\Results\Modeling\HADDOCK%20crosslinking-based%20models\divis_haddock_iaa07_2\divis_haddock_iaa07_2\cluster1_3.pdb) |  |
| Nr 4 best structure | [Download structure](file:///W:\05_FG_Signalintegration\Michael_Niemeyer\Results\Modeling\HADDOCK%20crosslinking-based%20models\divis_haddock_iaa07_2\divis_haddock_iaa07_2\cluster1_4.pdb) |  |

Cluster 2

| HADDOCK score | -76.7 +/- 7.5 |
| --- | --- |
| Cluster size | 39 |
| RMSD from the overall lowest-energy structure | 9.7 +/- 0.1 |
| Van der Waals energy | -81.6 +/- 9.2 |
| Electrostatic energy | -310.6 +/- 61.3 |
| Desolvation energy | 38.6 +/- 9.7 |
| Restraints violation energy | 284.7 +/- 23.34 |
| Buried Surface Area | 2658.3 +/- 106.6 |
| Z-Score | -1.0 |

| Nr 1 best structure | [Download structure](file:///W:\05_FG_Signalintegration\Michael_Niemeyer\Results\Modeling\HADDOCK%20crosslinking-based%20models\divis_haddock_iaa07_2\divis_haddock_iaa07_2\cluster2_1.pdb) | [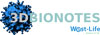](javascript:void(0)) |
| --- | --- | --- |
| Nr 2 best structure | [Download structure](file:///W:\05_FG_Signalintegration\Michael_Niemeyer\Results\Modeling\HADDOCK%20crosslinking-based%20models\divis_haddock_iaa07_2\divis_haddock_iaa07_2\cluster2_2.pdb) |  |
| Nr 3 best structure | [Download structure](file:///W:\05_FG_Signalintegration\Michael_Niemeyer\Results\Modeling\HADDOCK%20crosslinking-based%20models\divis_haddock_iaa07_2\divis_haddock_iaa07_2\cluster2_3.pdb) |  |
| Nr 4 best structure | [Download structure](file:///W:\05_FG_Signalintegration\Michael_Niemeyer\Results\Modeling\HADDOCK%20crosslinking-based%20models\divis_haddock_iaa07_2\divis_haddock_iaa07_2\cluster2_4.pdb) |  |

Cluster 3

| HADDOCK score | -57.9 +/- 9.1 |
| --- | --- |
| Cluster size | 24 |
| RMSD from the overall lowest-energy structure | 11.6 +/- 0.2 |
| Van der Waals energy | -59.2 +/- 5.7 |
| Electrostatic energy | -352.8 +/- 37.3 |
| Desolvation energy | 40.7 +/- 8.5 |
| Restraints violation energy | 311.3 +/- 103.62 |
| Buried Surface Area | 2191.0 +/- 60.8 |
| Z-Score | -0.1 |

| Nr 1 best structure | [Download structure](file:///W:\05_FG_Signalintegration\Michael_Niemeyer\Results\Modeling\HADDOCK%20crosslinking-based%20models\divis_haddock_iaa07_2\divis_haddock_iaa07_2\cluster3_1.pdb) | [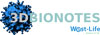](javascript:void(0)) |
| --- | --- | --- |
| Nr 2 best structure | [Download structure](file:///W:\05_FG_Signalintegration\Michael_Niemeyer\Results\Modeling\HADDOCK%20crosslinking-based%20models\divis_haddock_iaa07_2\divis_haddock_iaa07_2\cluster3_2.pdb) |  |
| Nr 3 best structure | [Download structure](file:///W:\05_FG_Signalintegration\Michael_Niemeyer\Results\Modeling\HADDOCK%20crosslinking-based%20models\divis_haddock_iaa07_2\divis_haddock_iaa07_2\cluster3_3.pdb) |  |
| Nr 4 best structure | [Download structure](file:///W:\05_FG_Signalintegration\Michael_Niemeyer\Results\Modeling\HADDOCK%20crosslinking-based%20models\divis_haddock_iaa07_2\divis_haddock_iaa07_2\cluster3_4.pdb) |  |

Cluster 5

| HADDOCK score | -57.3 +/- 9.3 |
| --- | --- |
| Cluster size | 17 |
| RMSD from the overall lowest-energy structure | 10.3 +/- 0.0 |
| Van der Waals energy | -74.7 +/- 6.6 |
| Electrostatic energy | -235.8 +/- 66.7 |
| Desolvation energy | 28.4 +/- 5.7 |
| Restraints violation energy | 361.0 +/- 29.05 |
| Buried Surface Area | 2532.4 +/- 51.5 |
| Z-Score | -0.0 |

| Nr 1 best structure | [Download structure](file:///W:\05_FG_Signalintegration\Michael_Niemeyer\Results\Modeling\HADDOCK%20crosslinking-based%20models\divis_haddock_iaa07_2\divis_haddock_iaa07_2\cluster5_1.pdb) | [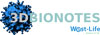](javascript:void(0)) |
| --- | --- | --- |
| Nr 2 best structure | [Download structure](file:///W:\05_FG_Signalintegration\Michael_Niemeyer\Results\Modeling\HADDOCK%20crosslinking-based%20models\divis_haddock_iaa07_2\divis_haddock_iaa07_2\cluster5_2.pdb) |  |
| Nr 3 best structure | [Download structure](file:///W:\05_FG_Signalintegration\Michael_Niemeyer\Results\Modeling\HADDOCK%20crosslinking-based%20models\divis_haddock_iaa07_2\divis_haddock_iaa07_2\cluster5_3.pdb) |  |
| Nr 4 best structure | [Download structure](file:///W:\05_FG_Signalintegration\Michael_Niemeyer\Results\Modeling\HADDOCK%20crosslinking-based%20models\divis_haddock_iaa07_2\divis_haddock_iaa07_2\cluster5_4.pdb) |  |

Cluster 9

| HADDOCK score | -56.7 +/- 14.6 |
| --- | --- |
| Cluster size | 6 |
| RMSD from the overall lowest-energy structure | 10.8 +/- 0.0 |
| Van der Waals energy | -54.3 +/- 5.1 |
| Electrostatic energy | -386.9 +/- 30.5 |
| Desolvation energy | 37.6 +/- 9.9 |
| Restraints violation energy | 374.3 +/- 92.60 |
| Buried Surface Area | 2154.1 +/- 158.5 |
| Z-Score | -0.0 |

| Nr 1 best structure | [Download structure](file:///W:\05_FG_Signalintegration\Michael_Niemeyer\Results\Modeling\HADDOCK%20crosslinking-based%20models\divis_haddock_iaa07_2\divis_haddock_iaa07_2\cluster9_1.pdb) | [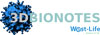](javascript:void(0)) |
| --- | --- | --- |
| Nr 2 best structure | [Download structure](file:///W:\05_FG_Signalintegration\Michael_Niemeyer\Results\Modeling\HADDOCK%20crosslinking-based%20models\divis_haddock_iaa07_2\divis_haddock_iaa07_2\cluster9_2.pdb) |  |
| Nr 3 best structure | [Download structure](file:///W:\05_FG_Signalintegration\Michael_Niemeyer\Results\Modeling\HADDOCK%20crosslinking-based%20models\divis_haddock_iaa07_2\divis_haddock_iaa07_2\cluster9_3.pdb) |  |
| Nr 4 best structure | [Download structure](file:///W:\05_FG_Signalintegration\Michael_Niemeyer\Results\Modeling\HADDOCK%20crosslinking-based%20models\divis_haddock_iaa07_2\divis_haddock_iaa07_2\cluster9_4.pdb) |  |

Cluster 4

| HADDOCK score | -55.2 +/- 15.0 |
| --- | --- |
| Cluster size | 19 |
| RMSD from the overall lowest-energy structure | 10.3 +/- 0.0 |
| Van der Waals energy | -77.9 +/- 16.6 |
| Electrostatic energy | -272.8 +/- 16.0 |
| Desolvation energy | 42.1 +/- 10.9 |
| Restraints violation energy | 351.8 +/- 70.65 |
| Buried Surface Area | 2622.3 +/- 263.2 |
| Z-Score | 0.0 |

| Nr 1 best structure | [Download structure](file:///W:\05_FG_Signalintegration\Michael_Niemeyer\Results\Modeling\HADDOCK%20crosslinking-based%20models\divis_haddock_iaa07_2\divis_haddock_iaa07_2\cluster4_1.pdb) | [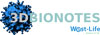](javascript:void(0)) |
| --- | --- | --- |
| Nr 2 best structure | [Download structure](file:///W:\05_FG_Signalintegration\Michael_Niemeyer\Results\Modeling\HADDOCK%20crosslinking-based%20models\divis_haddock_iaa07_2\divis_haddock_iaa07_2\cluster4_2.pdb) |  |
| Nr 3 best structure | [Download structure](file:///W:\05_FG_Signalintegration\Michael_Niemeyer\Results\Modeling\HADDOCK%20crosslinking-based%20models\divis_haddock_iaa07_2\divis_haddock_iaa07_2\cluster4_3.pdb) |  |
| Nr 4 best structure | [Download structure](file:///W:\05_FG_Signalintegration\Michael_Niemeyer\Results\Modeling\HADDOCK%20crosslinking-based%20models\divis_haddock_iaa07_2\divis_haddock_iaa07_2\cluster4_4.pdb) |  |

Cluster 8

| HADDOCK score | -22.9 +/- 5.7 |
| --- | --- |
| Cluster size | 7 |
| RMSD from the overall lowest-energy structure | 10.0 +/- 0.1 |
| Van der Waals energy | -54.9 +/- 3.4 |
| Electrostatic energy | -192.2 +/- 32.1 |
| Desolvation energy | 37.1 +/- 13.0 |
| Restraints violation energy | 333.9 +/- 53.49 |
| Buried Surface Area | 1972.6 +/- 171.1 |
| Z-Score | 1.6 |

| Nr 1 best structure | [Download structure](file:///W:\05_FG_Signalintegration\Michael_Niemeyer\Results\Modeling\HADDOCK%20crosslinking-based%20models\divis_haddock_iaa07_2\divis_haddock_iaa07_2\cluster8_1.pdb) | [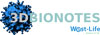](javascript:void(0)) |
| --- | --- | --- |
| Nr 2 best structure | [Download structure](file:///W:\05_FG_Signalintegration\Michael_Niemeyer\Results\Modeling\HADDOCK%20crosslinking-based%20models\divis_haddock_iaa07_2\divis_haddock_iaa07_2\cluster8_2.pdb) |  |
| Nr 3 best structure | [Download structure](file:///W:\05_FG_Signalintegration\Michael_Niemeyer\Results\Modeling\HADDOCK%20crosslinking-based%20models\divis_haddock_iaa07_2\divis_haddock_iaa07_2\cluster8_3.pdb) |  |
| Nr 4 best structure | [Download structure](file:///W:\05_FG_Signalintegration\Michael_Niemeyer\Results\Modeling\HADDOCK%20crosslinking-based%20models\divis_haddock_iaa07_2\divis_haddock_iaa07_2\cluster8_4.pdb) |  |

Cluster 7

| HADDOCK score | -19.9 +/- 8.6 |
| --- | --- |
| Cluster size | 10 |
| RMSD from the overall lowest-energy structure | 9.9 +/- 0.4 |
| Van der Waals energy | -53.1 +/- 3.9 |
| Electrostatic energy | -228.1 +/- 17.3 |
| Desolvation energy | 31.9 +/- 7.9 |
| Restraints violation energy | 469.8 +/- 61.38 |
| Buried Surface Area | 1889.2 +/- 143.1 |
| Z-Score | 1.7 |

| Nr 1 best structure | [Download structure](file:///W:\05_FG_Signalintegration\Michael_Niemeyer\Results\Modeling\HADDOCK%20crosslinking-based%20models\divis_haddock_iaa07_2\divis_haddock_iaa07_2\cluster7_1.pdb) | [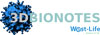](javascript:void(0)) |
| --- | --- | --- |
| Nr 2 best structure | [Download structure](file:///W:\05_FG_Signalintegration\Michael_Niemeyer\Results\Modeling\HADDOCK%20crosslinking-based%20models\divis_haddock_iaa07_2\divis_haddock_iaa07_2\cluster7_2.pdb) |  |
| Nr 3 best structure | [Download structure](file:///W:\05_FG_Signalintegration\Michael_Niemeyer\Results\Modeling\HADDOCK%20crosslinking-based%20models\divis_haddock_iaa07_2\divis_haddock_iaa07_2\cluster7_3.pdb) |  |
| Nr 4 best structure | [Download structure](file:///W:\05_FG_Signalintegration\Michael_Niemeyer\Results\Modeling\HADDOCK%20crosslinking-based%20models\divis_haddock_iaa07_2\divis_haddock_iaa07_2\cluster7_4.pdb) |  |

Results analysis

The results and graphics presented below are based on water-refined models generated by HADDOCK. The clusters (indicated in color in the graphs) are calculated based on the interface-ligand RMSDs calculated by HADDOCK, with the interface defined automatically based on all observed contacts. The various structural analysis [(FCC, i-RMSD and l-RMSD)](file:///W:\05_FG_Signalintegration\Michael_Niemeyer\Results\Modeling\HADDOCK%20crosslinking-based%20models\divis_haddock_iaa07_2\divis_haddock_iaa07_2\index.html#criteria) are made with respect to the best HADDOCK model (the one with the lowest HADDOCK score).

| [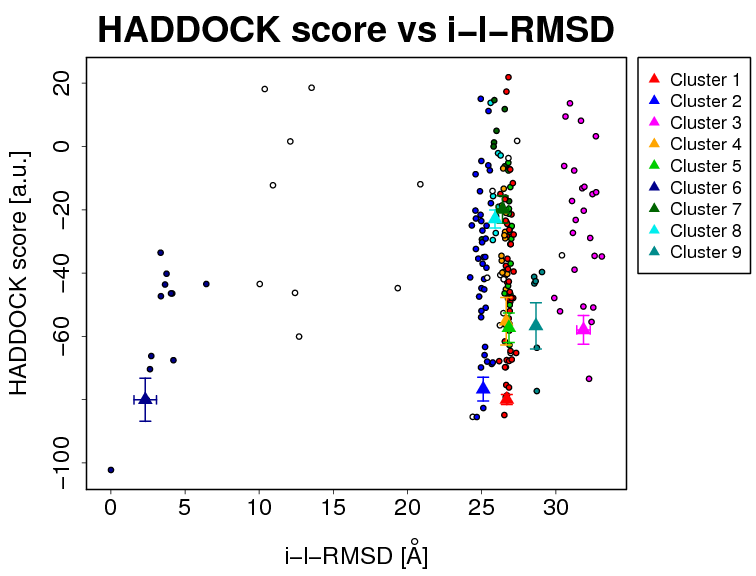](file:///\\isilon.ipb-halle.de\msv\05_FG_Signalintegration\Michael_Niemeyer\Results\Modeling\HADDOCK%20crosslinking-based%20models\divis_haddock_iaa07_2\divis_haddock_iaa07_2\ilrmsd_graph.png) | |
| --- | --- |
| [[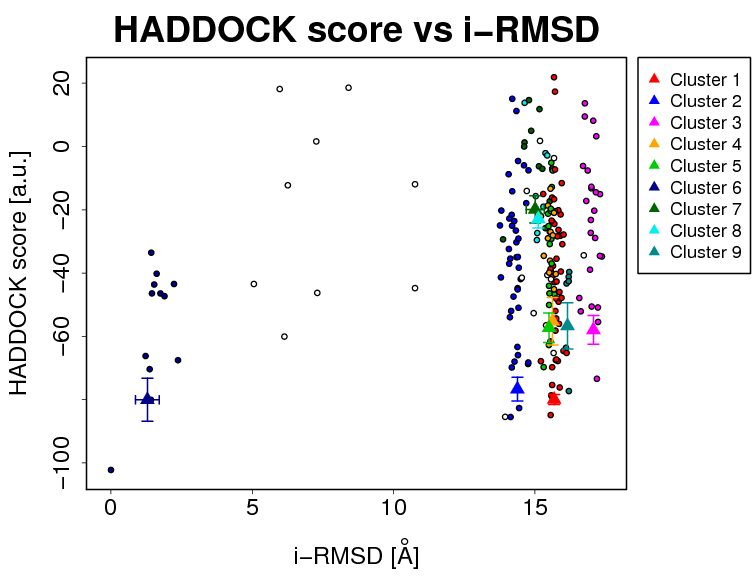](file:///\\isilon.ipb-halle.de\msv\05_FG_Signalintegration\Michael_Niemeyer\Results\Modeling\HADDOCK%20crosslinking-based%20models\divis_haddock_iaa07_2\divis_haddock_iaa07_2\irmsd_graph.png)](file:///W:\05_FG_Signalintegration\Michael_Niemeyer\Results\Modeling\HADDOCK%20crosslinking-based%20models\divis_haddock_iaa07_2\divis_haddock_iaa07_2\irmsd_graph.png) | [[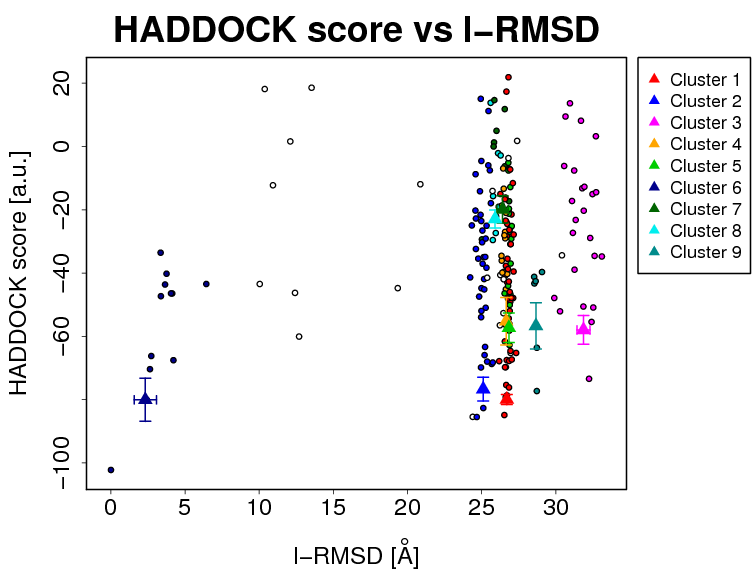](file:///\\isilon.ipb-halle.de\msv\05_FG_Signalintegration\Michael_Niemeyer\Results\Modeling\HADDOCK%20crosslinking-based%20models\divis_haddock_iaa07_2\divis_haddock_iaa07_2\lrmsd_graph.png)](file:///W:\05_FG_Signalintegration\Michael_Niemeyer\Results\Modeling\HADDOCK%20crosslinking-based%20models\divis_haddock_iaa07_2\divis_haddock_iaa07_2\lrmsd_graph.png) |
| [[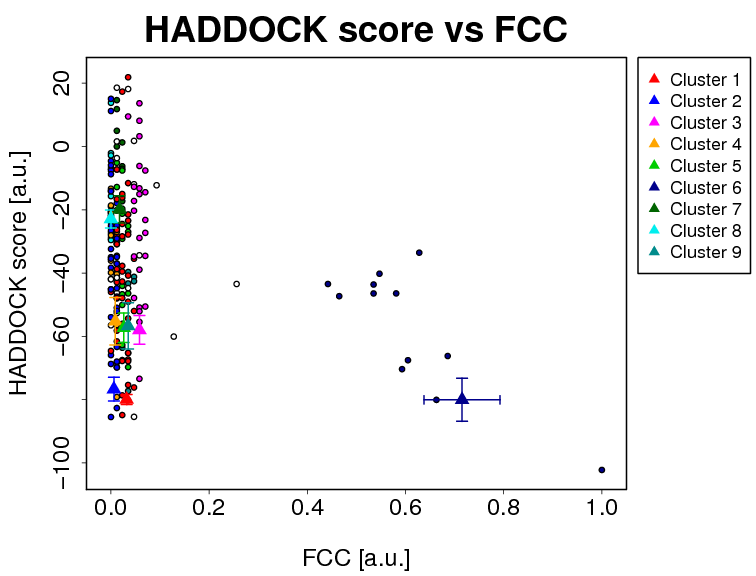](file:///\\isilon.ipb-halle.de\msv\05_FG_Signalintegration\Michael_Niemeyer\Results\Modeling\HADDOCK%20crosslinking-based%20models\divis_haddock_iaa07_2\divis_haddock_iaa07_2\fnat_graph.png)](file:///W:\05_FG_Signalintegration\Michael_Niemeyer\Results\Modeling\HADDOCK%20crosslinking-based%20models\divis_haddock_iaa07_2\divis_haddock_iaa07_2\fnat_graph.png) | [[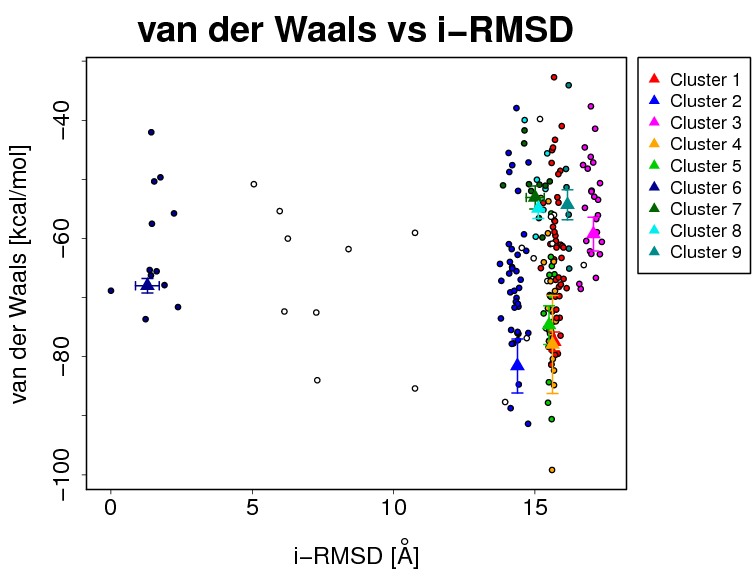](file:///\\isilon.ipb-halle.de\msv\05_FG_Signalintegration\Michael_Niemeyer\Results\Modeling\HADDOCK%20crosslinking-based%20models\divis_haddock_iaa07_2\divis_haddock_iaa07_2\vdw_graph.png)](file:///W:\05_FG_Signalintegration\Michael_Niemeyer\Results\Modeling\HADDOCK%20crosslinking-based%20models\divis_haddock_iaa07_2\divis_haddock_iaa07_2\vdw_graph.png) |
| [[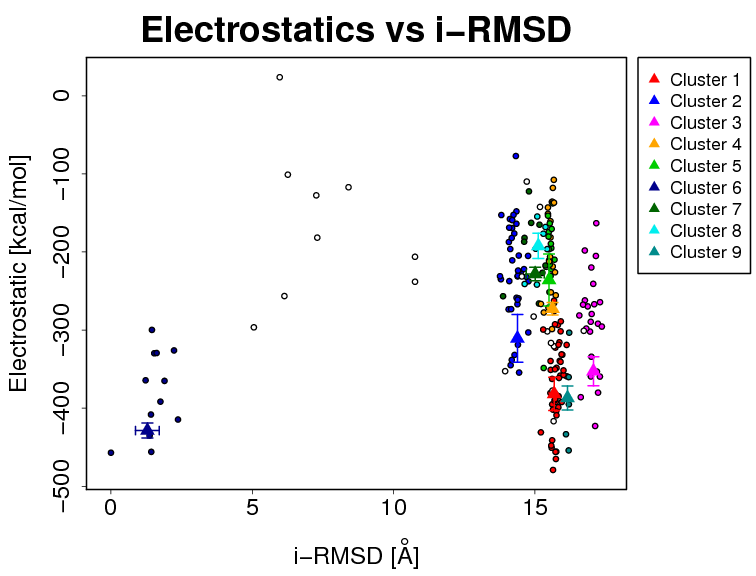](file:///\\isilon.ipb-halle.de\msv\05_FG_Signalintegration\Michael_Niemeyer\Results\Modeling\HADDOCK%20crosslinking-based%20models\divis_haddock_iaa07_2\divis_haddock_iaa07_2\elec_graph.png)](file:///W:\05_FG_Signalintegration\Michael_Niemeyer\Results\Modeling\HADDOCK%20crosslinking-based%20models\divis_haddock_iaa07_2\divis_haddock_iaa07_2\elec_graph.png) | [[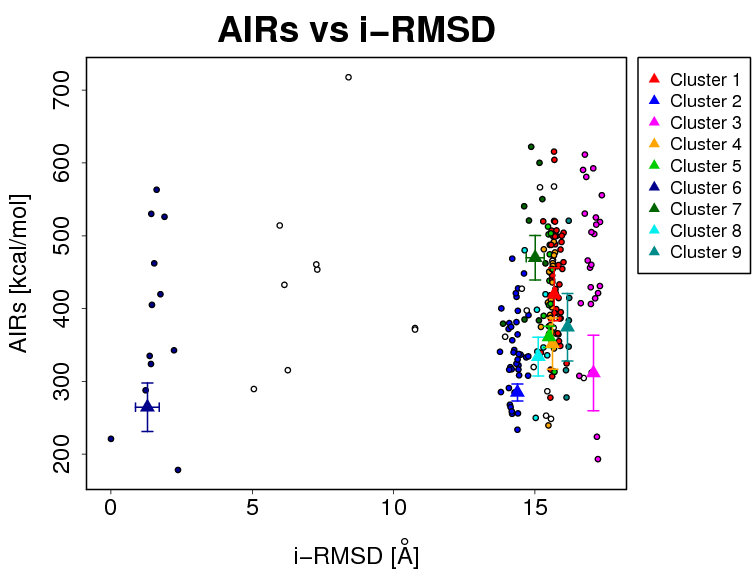](file:///\\isilon.ipb-halle.de\msv\05_FG_Signalintegration\Michael_Niemeyer\Results\Modeling\HADDOCK%20crosslinking-based%20models\divis_haddock_iaa07_2\divis_haddock_iaa07_2\air_graph.png)](file:///W:\05_FG_Signalintegration\Michael_Niemeyer\Results\Modeling\HADDOCK%20crosslinking-based%20models\divis_haddock_iaa07_2\divis_haddock_iaa07_2\air_graph.png) |

Supplementary information:

**i-RMSD** -> interface-RMSD calculated on the backbone (CA,C,N,O,P) atoms of all residues involved in intermolecular contact using a 10Å cutoff
**l-RMSD** -> ligand-RMSD calculated on the backbone atoms (CA,C,N,O,P) of all (N>1) molecules after fitting on the backbone atoms of the first (N=1) molecule
**FCC** -> Fraction of common contacts. The intermolecular contacts are defined based on the best HADDOCK model using a 5Å cutoff (see [Rodrigues et al, Proteins 2012](http://onlinelibrary.wiley.com/doi/10.1002/prot.24078/abstract))
**a.u.** -> Arbitrary Units
The cluster averages and standard deviations are indicated by colored dots with associated error bars. The average values are calculated on the best 4 structures of each clusters (based on the HADDOCK score).
