# Supplementary material for: Flexibility of intrinsically disordered degrons in AUX/IAA proteins reinforces auxin co-receptor assemblies
Source: Nat Commun. 2020 May 8;11:2277. doi: 10.1038/s41467-020-16147-2 (PMC7210949; doi:10.1038/s41467-020-16147-2)
Supplement: Supplementary file 11 — Supplementary Data 8 [file 41467_2020_16147_MOESM11_ESM.gz › disvis_haddock_files/IAA12/Summary.docx]

**HADDOCK server status for docking run /4156569441/divis_haddock_iaa12**

**Status: FINISHED**

Your HADDOCK run has successfully completed. The complete run can be downloaded as a gzipped tar file [**here**](http://milou.science.uu.nl/serviceresults/HADDOCK2.2/4156569441/divis_haddock_iaa12.tgz) (Note that there might be a delay in the generation of this archive - in case of errors try again). The file containing your docking parameters is [**here**](file:///W:\05_FG_Signalintegration\Michael_Niemeyer\Results\Modeling\HADDOCK%20crosslinking-based%20models\divis_haddock_iaa12\haddockparam.web).

Please cite the following papers in your work:

G.C.P van Zundert, J.P.G.L.M. Rodrigues, M. Trellet, C. Schmitz, P.L. Kastritis, E. Karaca, A.S.J. Melquiond, M. van Dijk, S.J. de Vries and A.M.J.J. Bonvin (2016). "[The HADDOCK2.2 webserver: User-friendly integrative modeling of biomolecular complexes](http://dx.doi.org/doi:10.1016/j.jmb.2015.09.014)."
*J. Mol. Biol.*, **428**, 720-725 (2015).

- Wassenaar *et al.*, [WeNMR: Structural Biology on the Grid.](http://link.springer.com/article/10.1007/s10723-012-9246-z)
  *J. Grid. Comp.*, **10**, 743-767 (2012).

And for the use of the WeNMR Grid resources please please add the following acknowledgement:
*"The FP7 [WeNMR](http://www.wenmr.eu) (project# 261572), H2020*[*West-Life*](http://www.west-life.eu)*(project# 675858) and the*[*EOSC-hub*](http://eosc-hub.eu)*(project# 777536) European e-Infrastructure projects are acknowledged for the use of their web portals, which make use of the*[*EGI*](http://www.egi.eu)*infrastructure with the dedicated support of CESNET-MCC, INFN-PADOVA, NCG-INGRID-PT, TW-NCHC, SURFsara and NIKHEF, and the additional support of the national GRID Initiatives of Belgium, France, Italy, Germany, the Netherlands, Poland, Portugal, Spain, UK, Taiwan and the US Open Science Grid."*

**How would you rate your experience with our portal?**sentiment_very_dissatisfied sentiment_dissatisfied sentiment_neutral sentiment_satisfied sentiment_very_satisfied

**Questions / feedback ?**[ask.bioexcel.eu](http://ask.bioexcel.eu)

**Announcing the 2020**[**EMBO practical course**](http://meetings.embo.org/event/20-biomolecular-interactions)**on Integrative Modelling of Biomolecular Interactions**.
It will take place at the Izmir Biomedicine and Genome Center (IBG), May 10-15, 2020.

**Summary**HADDOCK clustered**164**structures in**7**cluster(s), which represents**82.0 %**of the water-refined models HADDOCK generated. Note that currently the maximum number of models considered for clustering is 200.

**WARNING**: Clustering with default parameters did not produce any cluster, cluster minimum size went from **4** to
The statistics of the top 10 clusters are shown below. The top cluster is the most reliable according to HADDOCK. Its Z-score indicates how many standard deviations from the average this cluster is located in terms of score (the more negative the better).
A [graphical representation](file:///W:\05_FG_Signalintegration\Michael_Niemeyer\Results\Modeling\HADDOCK%20crosslinking-based%20models\divis_haddock_iaa12\index.html#graphics) of the results is also provided at the bottom of the page.

Cluster 4

| HADDOCK score | -116.9 +/- 12.2 |
| --- | --- |
| Cluster size | 9 |
| RMSD from the overall lowest-energy structure | 9.9 +/- 0.1 |
| Van der Waals energy | -78.0 +/- 2.5 |
| Electrostatic energy | -493.7 +/- 64.8 |
| Desolvation energy | 40.0 +/- 12.7 |
| Restraints violation energy | 199.6 +/- 57.94 |
| Buried Surface Area | 2683.9 +/- 96.9 |
| Z-Score | -1.2 |

| Nr 1 best structure | [Download structure](file:///W:\05_FG_Signalintegration\Michael_Niemeyer\Results\Modeling\HADDOCK%20crosslinking-based%20models\divis_haddock_iaa12\cluster4_1.pdb) | [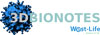](javascript:void(0)) |
| --- | --- | --- |
| Nr 2 best structure | [Download structure](file:///W:\05_FG_Signalintegration\Michael_Niemeyer\Results\Modeling\HADDOCK%20crosslinking-based%20models\divis_haddock_iaa12\cluster4_2.pdb) |  |
| Nr 3 best structure | [Download structure](file:///W:\05_FG_Signalintegration\Michael_Niemeyer\Results\Modeling\HADDOCK%20crosslinking-based%20models\divis_haddock_iaa12\cluster4_3.pdb) |  |
| Nr 4 best structure | [Download structure](file:///W:\05_FG_Signalintegration\Michael_Niemeyer\Results\Modeling\HADDOCK%20crosslinking-based%20models\divis_haddock_iaa12\cluster4_4.pdb) |  |

Cluster 3

| HADDOCK score | -114.6 +/- 14.0 |
| --- | --- |
| Cluster size | 10 |
| RMSD from the overall lowest-energy structure | 7.1 +/- 0.2 |
| Van der Waals energy | -62.6 +/- 4.5 |
| Electrostatic energy | -647.7 +/- 80.1 |
| Desolvation energy | 47.6 +/- 9.0 |
| Restraints violation energy | 298.6 +/- 52.19 |
| Buried Surface Area | 2466.5 +/- 159.7 |
| Z-Score | -1.1 |

| Nr 1 best structure | [Download structure](file:///W:\05_FG_Signalintegration\Michael_Niemeyer\Results\Modeling\HADDOCK%20crosslinking-based%20models\divis_haddock_iaa12\cluster3_1.pdb) | [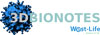](javascript:void(0)) |
| --- | --- | --- |
| Nr 2 best structure | [Download structure](file:///W:\05_FG_Signalintegration\Michael_Niemeyer\Results\Modeling\HADDOCK%20crosslinking-based%20models\divis_haddock_iaa12\cluster3_2.pdb) |  |
| Nr 3 best structure | [Download structure](file:///W:\05_FG_Signalintegration\Michael_Niemeyer\Results\Modeling\HADDOCK%20crosslinking-based%20models\divis_haddock_iaa12\cluster3_3.pdb) |  |
| Nr 4 best structure | [Download structure](file:///W:\05_FG_Signalintegration\Michael_Niemeyer\Results\Modeling\HADDOCK%20crosslinking-based%20models\divis_haddock_iaa12\cluster3_4.pdb) |  |

Cluster 2

| HADDOCK score | -99.0 +/- 6.2 |
| --- | --- |
| Cluster size | 37 |
| RMSD from the overall lowest-energy structure | 7.8 +/- 0.8 |
| Van der Waals energy | -66.2 +/- 6.1 |
| Electrostatic energy | -590.2 +/- 42.1 |
| Desolvation energy | 56.5 +/- 6.7 |
| Restraints violation energy | 286.9 +/- 26.97 |
| Buried Surface Area | 2227.2 +/- 36.3 |
| Z-Score | -0.5 |

| Nr 1 best structure | [Download structure](file:///W:\05_FG_Signalintegration\Michael_Niemeyer\Results\Modeling\HADDOCK%20crosslinking-based%20models\divis_haddock_iaa12\cluster2_1.pdb) | [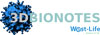](javascript:void(0)) |
| --- | --- | --- |
| Nr 2 best structure | [Download structure](file:///W:\05_FG_Signalintegration\Michael_Niemeyer\Results\Modeling\HADDOCK%20crosslinking-based%20models\divis_haddock_iaa12\cluster2_2.pdb) |  |
| Nr 3 best structure | [Download structure](file:///W:\05_FG_Signalintegration\Michael_Niemeyer\Results\Modeling\HADDOCK%20crosslinking-based%20models\divis_haddock_iaa12\cluster2_3.pdb) |  |
| Nr 4 best structure | [Download structure](file:///W:\05_FG_Signalintegration\Michael_Niemeyer\Results\Modeling\HADDOCK%20crosslinking-based%20models\divis_haddock_iaa12\cluster2_4.pdb) |  |

Cluster 6

| HADDOCK score | -94.2 +/- 15.4 |
| --- | --- |
| Cluster size | 7 |
| RMSD from the overall lowest-energy structure | 9.3 +/- 0.2 |
| Van der Waals energy | -66.0 +/- 7.8 |
| Electrostatic energy | -510.6 +/- 63.5 |
| Desolvation energy | 49.6 +/- 9.3 |
| Restraints violation energy | 243.0 +/- 101.00 |
| Buried Surface Area | 2439.0 +/- 134.8 |
| Z-Score | -0.3 |

| Nr 1 best structure | [Download structure](file:///W:\05_FG_Signalintegration\Michael_Niemeyer\Results\Modeling\HADDOCK%20crosslinking-based%20models\divis_haddock_iaa12\cluster6_1.pdb) | [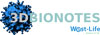](javascript:void(0)) |
| --- | --- | --- |
| Nr 2 best structure | [Download structure](file:///W:\05_FG_Signalintegration\Michael_Niemeyer\Results\Modeling\HADDOCK%20crosslinking-based%20models\divis_haddock_iaa12\cluster6_2.pdb) |  |
| Nr 3 best structure | [Download structure](file:///W:\05_FG_Signalintegration\Michael_Niemeyer\Results\Modeling\HADDOCK%20crosslinking-based%20models\divis_haddock_iaa12\cluster6_3.pdb) |  |
| Nr 4 best structure | [Download structure](file:///W:\05_FG_Signalintegration\Michael_Niemeyer\Results\Modeling\HADDOCK%20crosslinking-based%20models\divis_haddock_iaa12\cluster6_4.pdb) |  |

Cluster 1

| HADDOCK score | -71.0 +/- 6.4 |
| --- | --- |
| Cluster size | 90 |
| RMSD from the overall lowest-energy structure | 8.2 +/- 0.2 |
| Van der Waals energy | -68.8 +/- 10.1 |
| Electrostatic energy | -324.2 +/- 28.2 |
| Desolvation energy | 33.0 +/- 4.4 |
| Restraints violation energy | 296.8 +/- 42.87 |
| Buried Surface Area | 2022.3 +/- 43.8 |
| Z-Score | 0.7 |

| Nr 1 best structure | [Download structure](file:///W:\05_FG_Signalintegration\Michael_Niemeyer\Results\Modeling\HADDOCK%20crosslinking-based%20models\divis_haddock_iaa12\cluster1_1.pdb) | [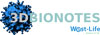](javascript:void(0)) |
| --- | --- | --- |
| Nr 2 best structure | [Download structure](file:///W:\05_FG_Signalintegration\Michael_Niemeyer\Results\Modeling\HADDOCK%20crosslinking-based%20models\divis_haddock_iaa12\cluster1_2.pdb) |  |
| Nr 3 best structure | [Download structure](file:///W:\05_FG_Signalintegration\Michael_Niemeyer\Results\Modeling\HADDOCK%20crosslinking-based%20models\divis_haddock_iaa12\cluster1_3.pdb) |  |
| Nr 4 best structure | [Download structure](file:///W:\05_FG_Signalintegration\Michael_Niemeyer\Results\Modeling\HADDOCK%20crosslinking-based%20models\divis_haddock_iaa12\cluster1_4.pdb) |  |

Cluster 7

| HADDOCK score | -67.2 +/- 24.1 |
| --- | --- |
| Cluster size | 4 |
| RMSD from the overall lowest-energy structure | 10.6 +/- 0.2 |
| Van der Waals energy | -64.8 +/- 11.2 |
| Electrostatic energy | -354.8 +/- 73.8 |
| Desolvation energy | 42.1 +/- 6.6 |
| Restraints violation energy | 264.2 +/- 51.25 |
| Buried Surface Area | 2409.1 +/- 107.4 |
| Z-Score | 0.8 |

| Nr 1 best structure | [Download structure](file:///W:\05_FG_Signalintegration\Michael_Niemeyer\Results\Modeling\HADDOCK%20crosslinking-based%20models\divis_haddock_iaa12\cluster7_1.pdb) | [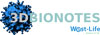](javascript:void(0)) |
| --- | --- | --- |
| Nr 2 best structure | [Download structure](file:///W:\05_FG_Signalintegration\Michael_Niemeyer\Results\Modeling\HADDOCK%20crosslinking-based%20models\divis_haddock_iaa12\cluster7_2.pdb) |  |
| Nr 3 best structure | [Download structure](file:///W:\05_FG_Signalintegration\Michael_Niemeyer\Results\Modeling\HADDOCK%20crosslinking-based%20models\divis_haddock_iaa12\cluster7_3.pdb) |  |
| Nr 4 best structure | [Download structure](file:///W:\05_FG_Signalintegration\Michael_Niemeyer\Results\Modeling\HADDOCK%20crosslinking-based%20models\divis_haddock_iaa12\cluster7_4.pdb) |  |

Cluster 5

| HADDOCK score | -45.7 +/- 14.2 |
| --- | --- |
| Cluster size | 7 |
| RMSD from the overall lowest-energy structure | 7.7 +/- 0.4 |
| Van der Waals energy | -65.0 +/- 5.2 |
| Electrostatic energy | -300.6 +/- 56.2 |
| Desolvation energy | 41.3 +/- 4.1 |
| Restraints violation energy | 381.0 +/- 45.13 |
| Buried Surface Area | 2047.6 +/- 130.9 |
| Z-Score | 1.7 |

| Nr 1 best structure | [Download structure](file:///W:\05_FG_Signalintegration\Michael_Niemeyer\Results\Modeling\HADDOCK%20crosslinking-based%20models\divis_haddock_iaa12\cluster5_1.pdb) | [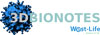](javascript:void(0)) |
| --- | --- | --- |
| Nr 2 best structure | [Download structure](file:///W:\05_FG_Signalintegration\Michael_Niemeyer\Results\Modeling\HADDOCK%20crosslinking-based%20models\divis_haddock_iaa12\cluster5_2.pdb) |  |
| Nr 3 best structure | [Download structure](file:///W:\05_FG_Signalintegration\Michael_Niemeyer\Results\Modeling\HADDOCK%20crosslinking-based%20models\divis_haddock_iaa12\cluster5_3.pdb) |  |
| Nr 4 best structure | [Download structure](file:///W:\05_FG_Signalintegration\Michael_Niemeyer\Results\Modeling\HADDOCK%20crosslinking-based%20models\divis_haddock_iaa12\cluster5_4.pdb) |  |

Results analysis

The results and graphics presented below are based on water-refined models generated by HADDOCK. The clusters (indicated in color in the graphs) are calculated based on the interface-ligand RMSDs calculated by HADDOCK, with the interface defined automatically based on all observed contacts. The various structural analysis [(FCC, i-RMSD and l-RMSD)](file:///W:\05_FG_Signalintegration\Michael_Niemeyer\Results\Modeling\HADDOCK%20crosslinking-based%20models\divis_haddock_iaa12\index.html#criteria) are made with respect to the best HADDOCK model (the one with the lowest HADDOCK score).

| [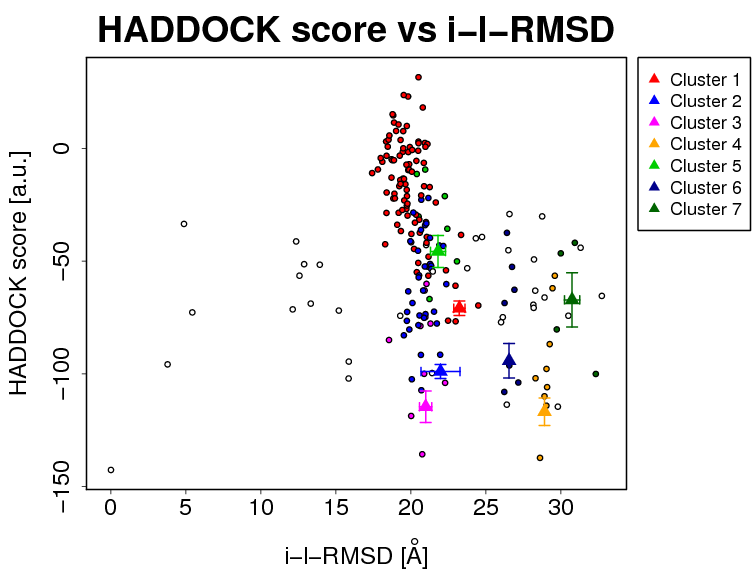](file:///\\isilon.ipb-halle.de\msv\05_FG_Signalintegration\Michael_Niemeyer\Results\Modeling\HADDOCK%20crosslinking-based%20models\divis_haddock_iaa12\ilrmsd_graph.png) | |
| --- | --- |
| [[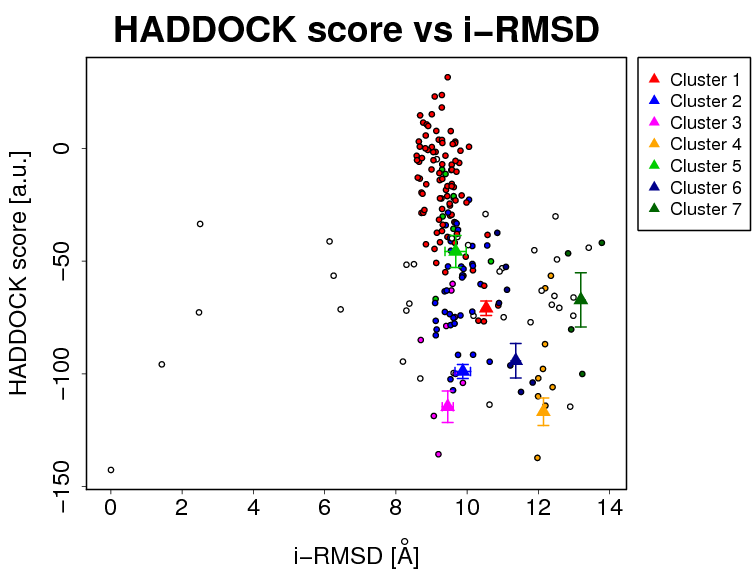](file:///\\isilon.ipb-halle.de\msv\05_FG_Signalintegration\Michael_Niemeyer\Results\Modeling\HADDOCK%20crosslinking-based%20models\divis_haddock_iaa12\irmsd_graph.png)](file:///W:\05_FG_Signalintegration\Michael_Niemeyer\Results\Modeling\HADDOCK%20crosslinking-based%20models\divis_haddock_iaa12\irmsd_graph.png) | [[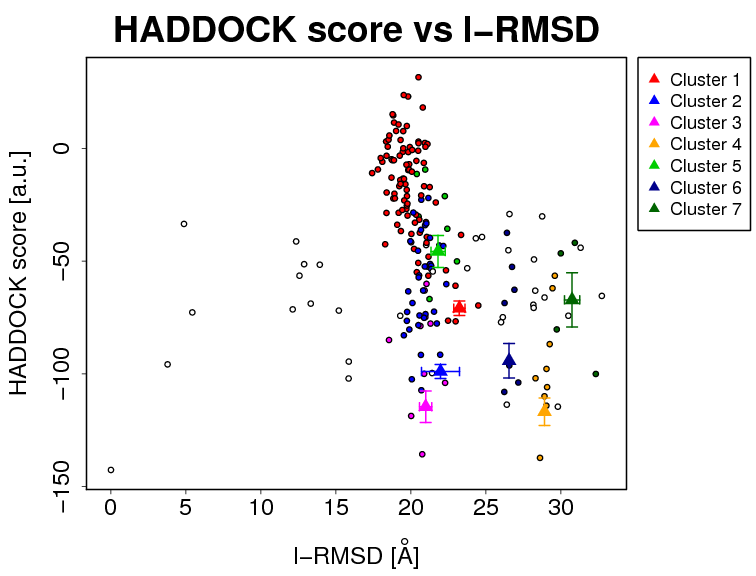](file:///\\isilon.ipb-halle.de\msv\05_FG_Signalintegration\Michael_Niemeyer\Results\Modeling\HADDOCK%20crosslinking-based%20models\divis_haddock_iaa12\lrmsd_graph.png)](file:///W:\05_FG_Signalintegration\Michael_Niemeyer\Results\Modeling\HADDOCK%20crosslinking-based%20models\divis_haddock_iaa12\lrmsd_graph.png) |
| [[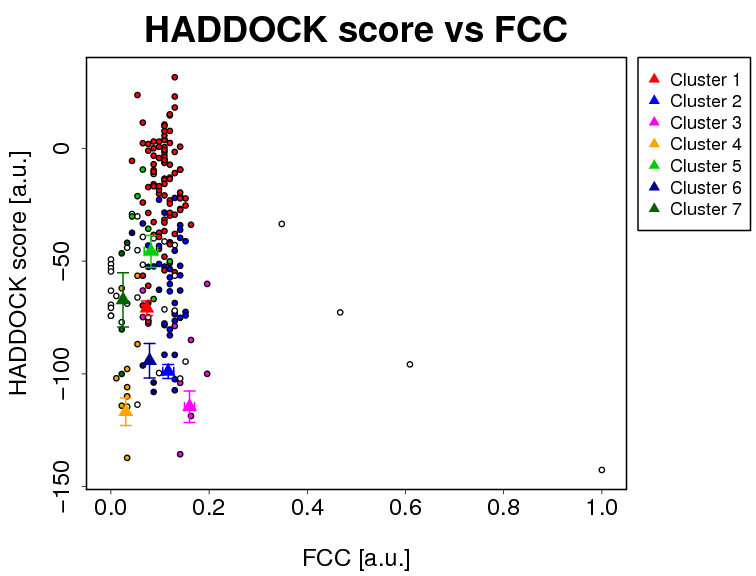](file:///\\isilon.ipb-halle.de\msv\05_FG_Signalintegration\Michael_Niemeyer\Results\Modeling\HADDOCK%20crosslinking-based%20models\divis_haddock_iaa12\fnat_graph.png)](file:///W:\05_FG_Signalintegration\Michael_Niemeyer\Results\Modeling\HADDOCK%20crosslinking-based%20models\divis_haddock_iaa12\fnat_graph.png) | [[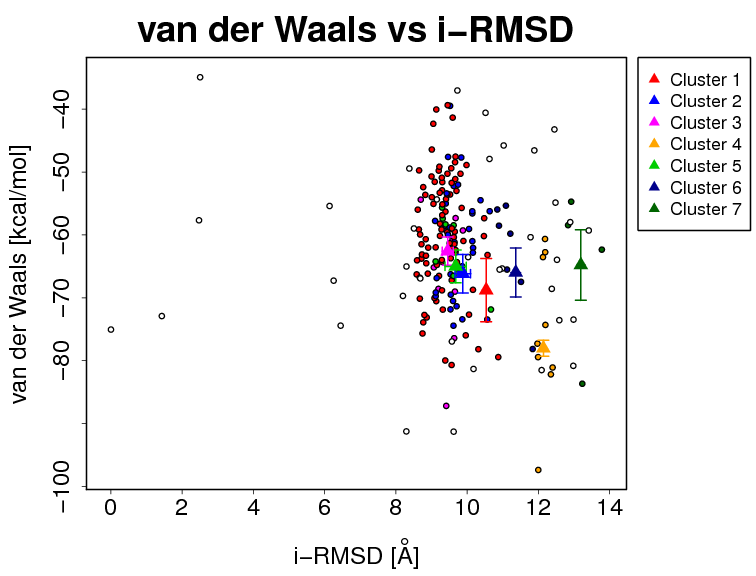](file:///\\isilon.ipb-halle.de\msv\05_FG_Signalintegration\Michael_Niemeyer\Results\Modeling\HADDOCK%20crosslinking-based%20models\divis_haddock_iaa12\vdw_graph.png)](file:///W:\05_FG_Signalintegration\Michael_Niemeyer\Results\Modeling\HADDOCK%20crosslinking-based%20models\divis_haddock_iaa12\vdw_graph.png) |
| [[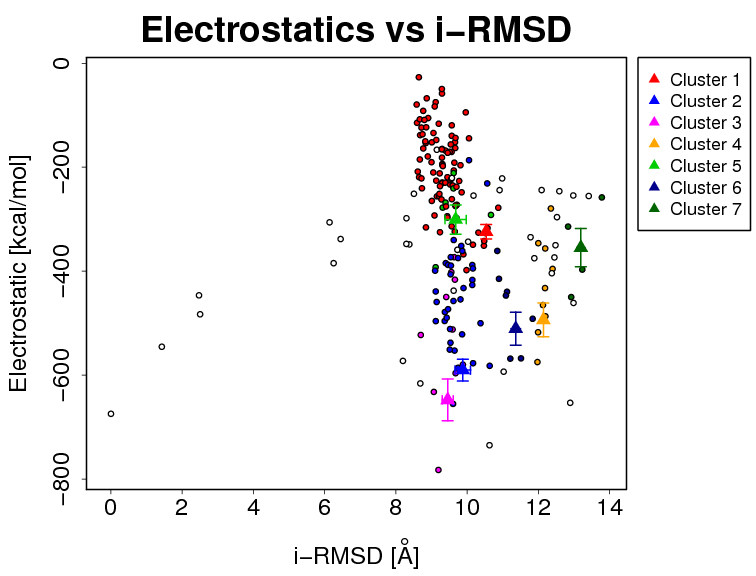](file:///\\isilon.ipb-halle.de\msv\05_FG_Signalintegration\Michael_Niemeyer\Results\Modeling\HADDOCK%20crosslinking-based%20models\divis_haddock_iaa12\elec_graph.png)](file:///W:\05_FG_Signalintegration\Michael_Niemeyer\Results\Modeling\HADDOCK%20crosslinking-based%20models\divis_haddock_iaa12\elec_graph.png) | [[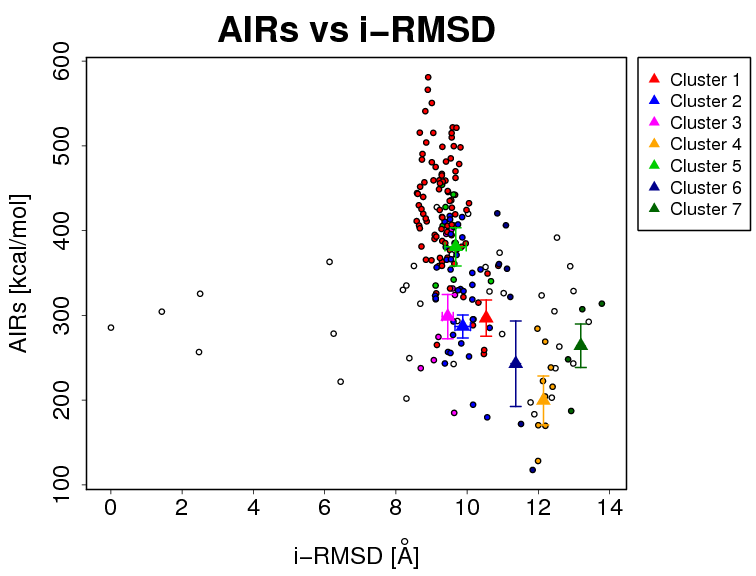](file:///\\isilon.ipb-halle.de\msv\05_FG_Signalintegration\Michael_Niemeyer\Results\Modeling\HADDOCK%20crosslinking-based%20models\divis_haddock_iaa12\air_graph.png)](file:///W:\05_FG_Signalintegration\Michael_Niemeyer\Results\Modeling\HADDOCK%20crosslinking-based%20models\divis_haddock_iaa12\air_graph.png) |

Supplementary information:

**i-RMSD** -> interface-RMSD calculated on the backbone (CA,C,N,O,P) atoms of all residues involved in intermolecular contact using a 10Å cutoff
**l-RMSD** -> ligand-RMSD calculated on the backbone atoms (CA,C,N,O,P) of all (N>1) molecules after fitting on the backbone atoms of the first (N=1) molecule
**FCC** -> Fraction of common contacts. The intermolecular contacts are defined based on the best HADDOCK model using a 5Å cutoff (see [Rodrigues et al, Proteins 2012](http://onlinelibrary.wiley.com/doi/10.1002/prot.24078/abstract))
**a.u.** -> Arbitrary Units
The cluster averages and standard deviations are indicated by colored dots with associated error bars. The average values are calculated on the best 4 structures of each clusters (based on the HADDOCK score).
