# Supplementary material for: Flexibility of intrinsically disordered degrons in AUX/IAA proteins reinforces auxin co-receptor assemblies
Source: Nat Commun. 2020 May 8;11:2277. doi: 10.1038/s41467-020-16147-2 (PMC7210949; doi:10.1038/s41467-020-16147-2)
Supplement: Supplementary file 13 — Reporting Summary [file 41467_2020_16147_MOESM13_ESM.pdf]

nature research

Corresponding author(s): Luz Irina A. Calderón Villalobos

Last updated by author(s): Apr 12, 2020

Reporting Summary

Nature Research wishes to improve the reproducibility of the work that we publish. This form provides structure for consistency and transparency in reporting. For further information on Nature Research policies, see Authors & Referees and the Editorial Policy Checklist .

Statistics

For all statistical analyses, confirm that the following items are present in the figure legend, table legend, main text, or Methods section.

n/a	Confirmed	
☐	☒	The exact sample size (*n*) for each experimental group/condition, given as a discrete number and unit of measurement
☐	☒	A statement on whether measurements were taken from distinct samples or whether the same sample was measured repeatedly
☐	☒	The statistical test(s) used AND whether they are one- or two-sided<br>*Only common tests should be described solely by name; describe more complex techniques in the Methods section.*
☒	☐	A description of all covariates tested
☐	☒	A description of any assumptions or corrections, such as tests of normality and adjustment for multiple comparisons
☐	☒	A full description of the statistical parameters including central tendency (e.g. means) or other basic estimates (e.g. regression coefficient) AND variation (e.g. standard deviation) or associated estimates of uncertainty (e.g. confidence intervals)
☐	☒	For null hypothesis testing, the test statistic (e.g. *F*, *t*, *r*) with confidence intervals, effect sizes, degrees of freedom and *P* value noted<br>*Give P values as exact values whenever suitable.*
☒	☐	For Bayesian analysis, information on the choice of priors and Markov chain Monte Carlo settings
☒	☐	For hierarchical and complex designs, identification of the appropriate level for tests and full reporting of outcomes
☒	☐	Estimates of effect sizes (e.g. Cohen's *d*, Pearson's *r*), indicating how they were calculated

Our web collection on statistics for biologists contains articles on many of the points above.

Software and code

Policy information about availability of computer code

Data collection	All information on softwares used in frame of this study has been included in the Methods section of the manuscript.
Data analysis	All information on data analyses implemented in this study have been included in the manuscript.

For manuscripts utilizing custom algorithms or software that are central to the research but not yet described in published literature, software must be made available to editors/reviewers. We strongly encourage code deposition in a community repository (e.g. GitHub). See the Nature Research guidelines for submitting code & software for further information.

Data

Policy information about availability of data

All manuscripts must include a data availability statement. This statement should provide the following information, where applicable:

- Accession codes, unique identifiers, or web links for publicly available datasets
- A list of figures that have associated raw data
- A description of any restrictions on data availability

All data used in the manuscript is either available in the Source Data File, via the respective repository entry or provided as separate files. Mass spectrometry proteomics data have been deposited to the ProteomeXchange Consortium via the PRIDE partner repository with the data sets identifiers: PXD015285 (XL-MS) and PXD015392 (ubiquitylation site identification data). All other information supporting the findings of this study is available from the corresponding author upon reasonable request.

Field-specific reporting

Please select the one below that is the best fit for your research. If you are not sure, read the appropriate sections before making your selection.

Life sciences study design

All studies must disclose on these points even when the disclosure is negative.

Sample size	Sample size calculations were not performed. Sample sizes were adjusted for reasonable handling and sufficient validity.
Data exclusions	One IAA concentration was omitted from one replica in auxin bindings due to an obvious pipetting error. Raw is marked in Source data file.
Replication	All experiments, if not otherwise stated were performed at least 3 times. Most were performed more than 3 times. Detailed information can be found in the corresponding figure legends.
Randomization	Processing of samples was randomized. MS measurements were performed by a different person, than the one prepared the samples.
Blinding	Most experiments were performed by a single person. MS measurement differ (see above).

Reporting for specific materials, systems and methods

We require information from authors about some types of materials, experimental systems and methods used in many studies. Here, indicate whether each material, system or method listed is relevant to your study. If you are not sure if a list item applies to your research, read the appropriate section before selecting a response.

Materials & experimental systems

n/a	Involved in the study	
☐	☒	Antibodies
☒	☐	Eukaryotic cell lines
☒	☐	Palaeontology
☐	☒	Animals and other organisms
☒	☐	Human research participants
☒	☐	Clinical data

Methods

n/a	Involved in the study	
☒	☐	ChIP-seq
☒	☐	Flow cytometry
☒	☐	MRI-based neuroimaging

Antibodies

Antibodies used

Secondary anti-rabbit Alexa Fluor® Plus 647 antibody (Thermo Fischer Scientific, A32733)
Primary polyclonal anti-GST in rabbit (Sigma Aldrich, G7781)
Primary anti-HA(F-7): Santa Cruz Biotechnology (sc-7392),
Primary anti LexA: abcam (ab14553),
Primary anti-Tubulin (YL1/2): abcam (ab6160),
Secondary anti-rabbit-AP: Sigma-Aldrich (A3687),
Secondary anti-mouse-AP: Sigma-Aldrich (A2179).

Validation

Primary anti-Tubulin (YL1/2): abcam (ab6160) from: https://www.abcam.com/tubulin-antibody-yl12-loading-control-ab6160.html
Description
Rat monoclonal [YL1/2] to Tubulin - Loading Control
Host species
Rat
Specificity
The YL1/2 monoclonal has been reported in the literature to recognise tyrosinated alpha tubulin but can also bind other targets that contain a negatively charged carboxy terminus such as recA and oxidized actin (PubMed IDs: 6811596, 6415068, 6204858). The amino acid sequence Glu-Glu-Phe (EEF) has been used as a carboxy terminus tag to purify proteins using the YL1/2 monoclonal (PubMed IDs: 1710580, 1713577).

Tested applications
Suitable for: ELISA, IHC-Fr, IP, RIA, WB, Flow Cyt, ICC/IF, IHC (PFA fixed), IHC-P, IHC - Wholemount, IHC (Methanol fixed)more details
Species reactivity
Reacts with: Mouse, Human, Pig, Saccharomyces cerevisiae, Xenopus laevis, Caenorhabditis elegans, Drosophila melanogaster, Schizosaccharomyces pombe, a wide range of other species, Mammals, African green monkey

Primary polyclonal anti-GST in rabbit (Sigma Aldrich, G7781) from: https://www.sigmaaldrich.com/catalog/product/sigma/g7781?lang=de&region=DE
General description

Anti-Glutathione-S-Transferase (GST) antibody produced in rabbit is specific for native as well as denatured-reduced forms of glutathione-S-transferase from Schistosoma japonicum. The soluble GST is encoded by eight different genes in humans namely, α, μ, θ, π, ζ, σ, κ and ω on chromosome 6, 1, 22, 11, 14, 4, not known and 10 respectively.[3]

The antibody is specific for native as well as denatured-reduced forms of glutathione-S-transferase from Schistosoma japonicum. Anti-GST may be used in various immunoassays to identify the expression of GST fusion proteins.

Primary anti-HA(F-7): Santa Cruz Biotechnology (sc-7392) from: hhttps://datasheets.scbt.com/sc-7392.pdf
APPLICATIONS
HA-probe (F-7) is recommended for detection of proteins containing the HA tag by Western Blotting (starting dilution 1:200, dilution range 1:100-1:1000), immunoprecipitation [1-2 µg per 100-500 µg of total protein (1 ml of cell lysate)], immunofluorescence (starting dilution 1:50, dilution range 1:50-1:500), [...]

Primary anti LexA: abcam (ab14553) from: https://www.abcam.com/lexa-dna-binding-region-antibody-ab14553.html
Description
Rabbit polyclonal to LexA DNA Binding Region
Host species
Rabbit
Specificity
Recognizes the LexA portion of bait fusion proteins used in the yeast two-hybrid system.
Tested applications
Suitable for: WB, IPmore details
Species reactivity
Reacts with: Species independent

Animals and other organisms

Policy information about studies involving animals; ARRIVE guidelines recommended for reporting animal research

Laboratory animals	No animals were used in this study.<br>Plants (Arabidopsis thaliana) either Col-0 wild-type, or tir1-1 mutants were used.<br>tir1-1 mutant: Identified in an EMS-based screen for resistance to auxin-transport inhibitors (Ruegger et al. 1997 & 1998)<br>Plants were either grown under continuous light or long day conditions (16h day, 8h night, at 22°C)
Wild animals	No wild animals were used in this study
Field-collected samples	No field-collected samples were used in this study
Ethics oversight	No ethical approval was required for the conducted plant work

Note that full information on the approval of the study protocol must also be provided in the manuscript.
